# Recent advances in poly(butylene adipate-*co*-terephthalate) (PBAT): structure, modification strategies, degradation, and sustainable applications

**DOI:** 10.3389/fchem.2026.1862076

**Published:** 2026-07-28

**Authors:** Xinyue Wang, Jintang Guo

**Affiliations:** School of Chemical Engineering and Technology, Tianjin University, Tianjin, China

**Keywords:** blending, degradation, PBAT, polyester, sustainable applications

## Abstract

Poly (butylene adipate-*co*-terephthalate) (PBAT), a biodegradable copolyester, integrates the pliability of aliphatic chains with the rigidity and durability of aromatic components, emerging as a compelling substitute for traditional petroleum-derived plastics. This review summarizes the advances in the structure, physicochemical properties, and degradation behavior of PBAT, with particular emphasis on modification strategies through blending and composite design. Blending PBAT with biodegradable polymers, as well as natural macromolecules, has been widely explored to improve its mechanical properties, processability, and barrier performance without sacrificing its inherent full biodegradable characteristic. The incorporation of inorganic fillers and nanomaterials further enhances the thermal stability and functional performance of PBAT-based composites. PBAT exhibits effective biodegradation in soil, composting, and aquatic environments, contributing to the reduction of plastic pollution. Owing to these advantages, PBAT has attracted increasing attention in applications ranging from agricultural mulching films, packaging materials, biomedical scaffolds, to textile fibers. Nevertheless, challenges including high production costs, strict degradation conditions, and the potential formation of microplastics during degradation still threaten the ecological environment. Future research should focus on developing catalytic synthesis routes, optimizing copolymer compositions and blends, and advancing enzyme-assisted and chemical recycling to promote closed-loop material utilization. The practical applications of PBAT are expected to be further expanded by these efforts, thereby supporting the transition toward a more sustainable and circular polymer economy.

## 1 Introduction

Plastic pollution has become a global environmental issue (MacLeod et al., 2021). Microscopic plastic debris, known as microplastics (MPs), is increasingly detected in various ecosystems (Thompson et al., 2024). Traditional non-biodegradable plastics, such as polyethylene, polypropylene, and polystyrene, persist in the environment for hundreds of years, causing long-term ecological damage (Napper and Thompson, 2020). These plastics accumulate in landfills, contaminate water bodies, and even threaten wildlife (Gunaalan et al., 2020; Sun et al., 2024). For instance, plastic debris in marine environments can entangle marine animals, cause ingestion problems, and disrupt the food chain (Tuuri and Leterme, 2023). The prevalence of MPs throughout the global food chain has sparked widespread concern over their potential adverse impacts on human health (Mamun et al., 2023). Agricultural activities also significantly aggravate plastic pollution, particularly through the widespread use of plastic mulch films. These films, typically made of non-biodegradable plastics, will remain in the soil after use, leading to soil degradation and reduce crop productivity (Campanale et al., 2024). Moreover, the improper disposal of packaging materials, including single-use plastic bags and food containers, further aggravates plastic pollution (Nayanathara Thathsarani Pilapitiya and Ratnayake, 2024). The development of sustainable alternatives has become increasingly important, driving extensive research on biodegradable polymers. Biodegradable polymers offer the potential to reduce plastic waste and mitigate environmental impacts, as they can be decomposed by microorganisms into natural substances under specific environmental conditions (Afshar et al., 2024).

Aliphatic polyesters have received particular attention in the development of biodegradable plastics because they can be readily degraded by microorganisms. Typical examples include polycaprolactone (PCL), poly β-hydroxybutyrate (PHB), and polybutylene succinate (PBS). Although these polymers exhibit good biodegradability, they generally have poor mechanical strength and limited thermal stability (Al Hosni et al., 2019; De Falco et al., 2021; Lee and Salleh, 2025; McAdam et al., 2020; Shaiju et al., 2020). By contrast, aromatic polyesters including polyethylene terephthalate and polybutylene terephthalate exhibit exceptional mechanical strength and thermal stability, yet exhibit extreme recalcitrance to biodegradation in natural environments (Dhaka et al., 2022; Qiu et al., 2024). Poly (butylene adipate-*co*-terephthalate) (PBAT) has emerged as an important biodegradable copolyester that combines the advantages of both aliphatic and aromatic polyesters (Yang Y. et al., 2023). The aliphatic segments in PBAT provide flexibility and biodegradability, while the aromatic segments improve mechanical strength and thermal stability (Kumar et al., 2010; Pavon et al., 2020).

This review summarizes the current knowledge on PBAT, including its chemical structure, properties, synthesis methods, degradation behavior, and applications. It also identifies key gaps in current research and outlines promising future research directions. These insights may help guide the development of more efficient and sustainable PBAT-based materials, contributing to the reduction of plastic pollution and the advancement of a circular economy.

## 2 Chemical structure and composition of PBAT

**FIGURE 1:**
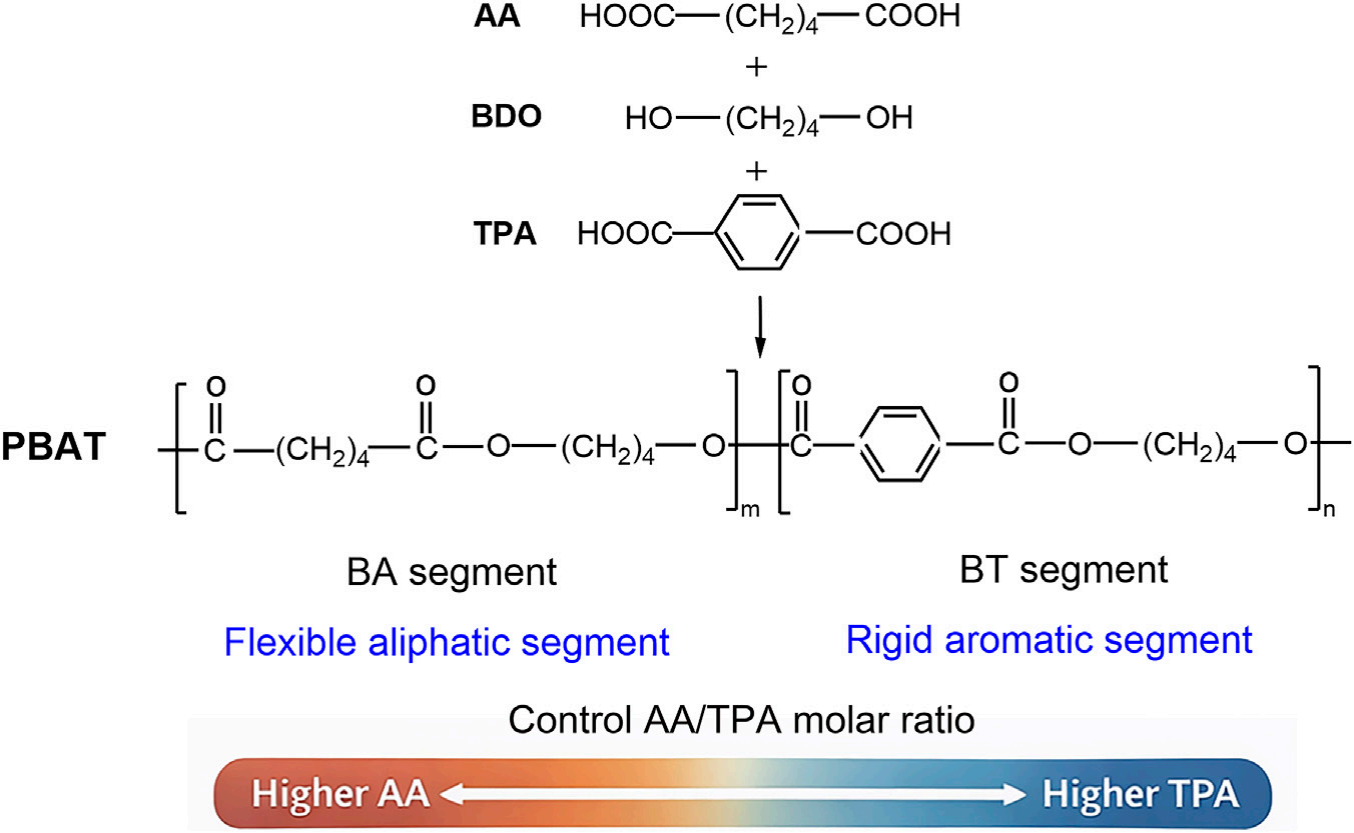
Structural architecture of PBAT.

PBAT is composed of adipic acid (AA), terephthalic acid (TPA), and 1,4-butanediol (BDO) monomers (Zheng et al., 2024). PBAT is typically synthesized through a polycondensation reaction. During this process, the carboxyl groups of AA and TPA react with the hydroxyl groups of BDO to form ester linkages and release water. The ratio of AA to TPA in PBAT plays an important role in determining the crystallinity and mechanical properties of the copolymer (Wang J. et al., 2024). A higher AA content generally increases the flexibility of the PBAT chain. Conversely, a higher TPA content tends to produce a more rigid polymer structure (Figure 1).

As a result, the monomer ratio can significantly influence the tensile strength, elongation at break, and melting temperature of PBAT. Additionally, the comonomer ratio can influence the biodegradability of PBAT, as different ratios may affect the accessibility of the polymer chain to enzymes or other degradation agents (Jian et al., 2020). Understanding the chemical structure and composition of PBAT is essential for explaining its synthesis, properties, blending behavior, and applications.

## 3 Synthesis of PBAT

**FIGURE 2:**
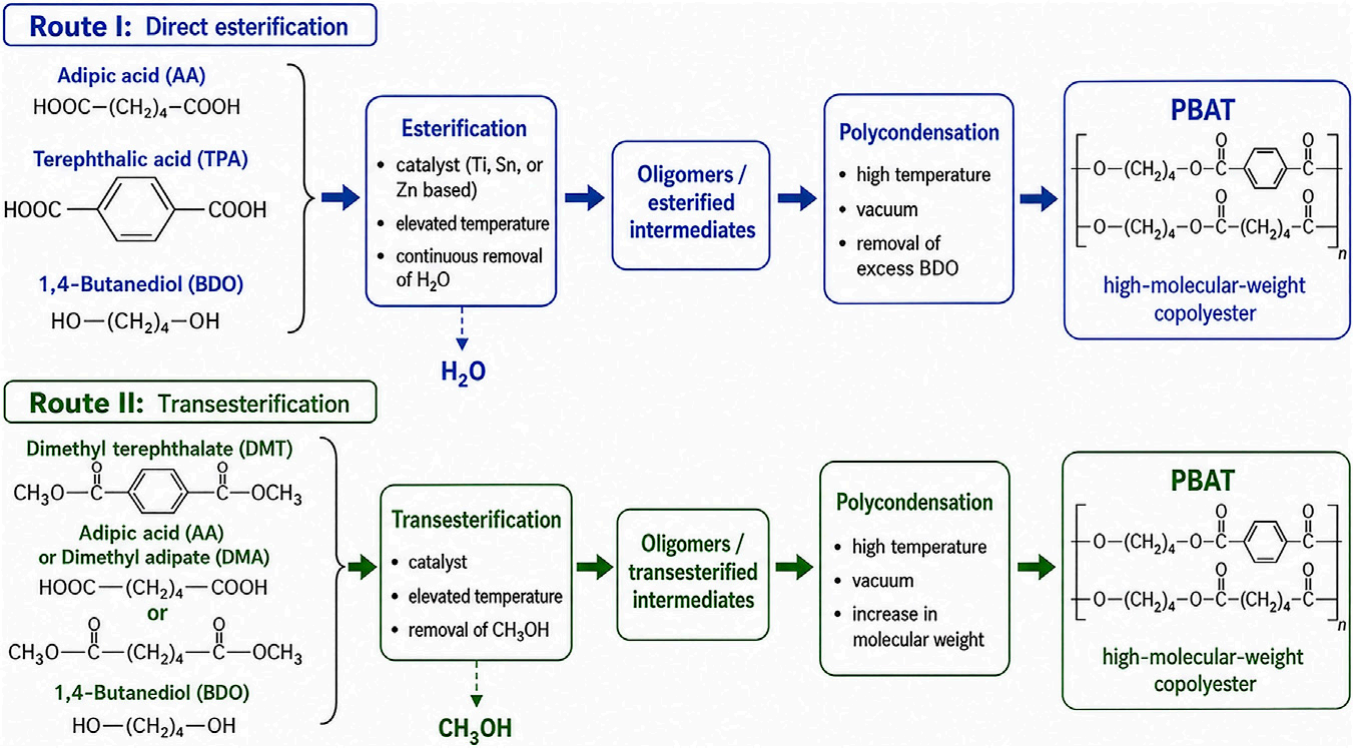
Schematic illustration of the two main synthesis routes of PBAT.

Two common synthesis routes for PBAT are direct esterification and transesterification, as schematically illustrated in Figure 2. PBAT is commonly synthesized through melt polycondensation using BDO, AA, and TPA or their ester derivatives as monomers. In direct esterification, AA, TPA, and BDO react directly in the presence of a catalyst to form PBAT. In this route, AA, TPA, and excess BDO are first reacted in the presence of catalysts, such as titanium-, tin-, or zinc-based compounds, to form esterified oligomers. Water generated during esterification is continuously removed to drive the reaction forward. The resulting oligomers are then subjected to high-temperature and reduced-pressure polycondensation to remove excess BDO and increase the molecular weight of PBAT(Jian et al., 2020; Kathula et al., 2025). This method is relatively simple because it does not require additional ester intermediates. However, high reaction temperatures and longer reaction time are usually required to achieve a higher conversion rate (Lee et al., 2024). In contrast, transesterification involves the reaction of dimethyl terephthalate (DMT) and dimethyl adipate or adipic acid with BDO. In this route, DMT and dimethyl adipate or adipic acid are reacted with BDO in the presence of a catalyst. Transesterification occurs between ester groups and hydroxyl groups, producing low-molecular-weight oligomers or prepolymers and releasing methanol as a by-product when DMT or dimethyl adipate is used (Jian et al., 2020). The initial reaction produces oligomers or prepolymers, which are subsequently subjected to polycondensation to form PBAT. The oligomers are then subjected to further polycondensation under vacuum to obtain high-molecular-weight PBAT. Compared with direct esterification, transesterification can be carried out at relatively lower temperatures, which may reduce energy consumption and minimize side reactions (Cornu et al., 2015). These two routes represent the most widely used methods for PBAT production in both laboratory research and industrial manufacturing.

Both synthesis routes have their own advantages and limitations, and the suitable method depends on factors such as product quality, production scale, and cost. In industrial production, transesterification is often considered suitable for large-scale manufacturing because it allows better control of reaction conditions and polymer quality. However, direct esterification may still be preferred in certain cases due to its simpler process and lower production cost. Overall, both routes include an esterification or transesterification stage followed by a polycondensation stage. The final molecular weight, copolymer composition, crystallinity, thermal behavior, mechanical properties, and biodegradability of PBAT are strongly affected by the AA/TPA ratio, BDO excess, catalyst type, reaction temperature, reaction time, and vacuum conditions. Therefore, optimization of synthesis conditions is essential for producing PBAT with balanced flexibility, thermal stability, mechanical strength, and biodegradability (Oliver-Cuenca et al., 2024).

In polymer synthesis, including PBAT production, polycondensation can generally be classified into one-step and two-step processes. In the one-step process, all monomers are introduced into the reactor simultaneously, and polymerization occurs in a single stage. This approach is relatively efficient in terms of processing time and equipment requirements, although precise control of the reaction may be more challenging (Su et al., 2021). In the two-step process, a prepolymerization stage followed by a final polycondensation step enables more accurate control of molecular weight and polymer structure but often requires more complex process management (Parcheta and Datta, 2020). Currently, one-step polycondensation is widely used because it can effectively promote the co-condensation of AA and TPA and control the copolymer composition to obtain polymers with balanced performance (Kathula et al., 2025). In contrast, two-step polycondensation offers limited advantages in terms of cost and operational complexity and is therefore mainly applied in laboratory studies or functional modification research (Zhang X. et al., 2025).

## 4 Properties of PBAT

### 4.1 Mechanical properties of PBAT

The mechanical performance of PBAT is analogous to that of low-density polyethylene (LDPE) (Küster et al., 2025). Its wide application is closely related to its superior flexibility, high elongation at break, and favorable processability. These properties are strongly influenced by the molecular weight of PBAT. In the PBAT molecular structure, BDO and AA form flexible aliphatic segments, commonly referred to as BA segments, which provide high flexibility to the polymer chain (Myalenko and Fedotova, 2023). As a result, PBAT typically exhibits higher elongation at break than many other biodegradable plastics (Chen et al., 2025). In contrast, the aromatic segments formed by BDO and TPA, known as BT segments, act as rigid structural units, and the mechanical properties of PBAT are strongly influenced by the content of these segments. Increasing the BT content generally enhances the Young’s modulus of the polymer but reduces the elongation at break (Herrera et al., 2002). When maintaining the biodegradability of PBAT while improving its mechanical strength, the maximum TPA content can reach approximately 40 wt% (Küster et al., 2025).

Generally, tensile strength increases with increasing molecular weight, whereas elongation at break tends to decrease (Jian et al., 2020). Therefore, during polymer production, parameters such as reaction temperature, pressure, and catalyst concentration can be adjusted to control the molecular weight and thereby tune the mechanical properties of PBAT (Ferreira et al., 2019). In addition, the mechanical performance of PBAT-based materials can be further improved by incorporating fillers. The addition of fillers can reduce production costs while maintaining biodegradability and enhancing mechanical properties.

### 4.2 Thermal properties and stability of PBAT

Because polymer chain contains both flexible aliphatic segments and rigid aromatic terephthalate units, PBAT maintains good flexibility while exhibiting relatively high thermal stability. Differential scanning calorimetry (DSC) analysis shows that PBAT has a glass transition temperature (T_g_) of approximately −30 °C, while its melting temperature (T_m_) typically ranges from 110 °C–130 °C (Garalde et al., 2019; Sui et al., 2023; Tang et al., 2024). Thermogravimetric analysis (TGA) indicates that the initial decomposition temperature (T_onset_) of PBAT is generally between 330 °C and 360 °C, with the main degradation stage occurring between 370 °C and 430 °C. The residual carbon content after thermal degradation is typically around 2%–5%, indicating relatively good thermal stability (Grimaut et al., 2023; Kanwal et al., 2022b). The thermal degradation of PBAT generally follows a two-stage mechanism. The first stage involves the cleavage of aliphatic ester bonds, while the second stage includes the fragmentation and rearrangement of aromatic segments (Perumal et al., 2024). Due to its relatively high thermal stability, PBAT can maintain structural integrity during common processing operations such as melt extrusion, film blowing, and hot pressing. This stability allows PBAT to retain its mechanical and physical properties during high-temperature processing and storage.

### 4.3 Biodegradability of PBAT

PBAT degradation generally involves several sequential steps, including microbial colonization and biofilm formation on the polymer surface, secretion of extracellular enzymes, enzymatic hydrolysis of ester bonds in the PBAT backbone, formation of low-molecular-weight oligomers and monomers, microbial assimilation of degradation products, and final mineralization into CO_2_, H_2_O, and microbial biomass under aerobic conditions. The aliphatic adipate units in PBAT are generally more susceptible to enzymatic cleavage, whereas the aromatic terephthalate units show higher resistance, which may slow the overall degradation process (Fernandes et al., 2025). Several microorganisms have been reported to participate in PBAT degradation through enzyme-mediated mechanisms. Bacterial genera such as *Pseudomonas* and *Bacillus* can attach to the PBAT surface and secrete extracellular esterases or lipases, which hydrolyze ester bonds and promote polymer chain scission (Kanwal et al., 2022a; Santos-Beneit et al., 2023). Actinomycetes, including *Streptomyces*, *Amycolatopsis*, *Thermobifida*, and *Thermomonospora*, have been reported to contribute to PBAT degradation or hydrolysis. These microorganisms may produce PBAT-degrading esterases, cutinase-like enzymes, or polyester hydrolases, thereby facilitating the breakdown of PBAT into smaller biodegradable fragments (Rüthi et al., 2023). Fungal microorganisms can also participate in PBAT biodegradation through enzyme-mediated depolymerization. For example, *Fusarium solani* cutinase has been shown to hydrolyze PBAT (Lin et al., 2023), while fungal strains such as *Purpureocillium lilacinum* have also been reported to show PBAT hydrolysis potential (Tseng et al., 2023). These extracellular enzymes, including cutinases, esterases, and lipases, can adsorb onto the polymer surface, hydrolyze ester bonds, reduce polymer molecular weight, and generate oligomers or monomers that are subsequently assimilated and metabolized by microorganisms.

As a biodegradable polyester, the degradation behavior of PBAT in various environments has attracted significant research attention (Oh et al., 2025). In soil environments, Zumstein et al. demonstrated using ^13^C-labeled PBAT and isotope-specific analysis that PBAT can be degraded by soil microorganisms, and the carbon from the polymer can be converted into CO_2_ and microbial biomass (Zumstein et al., 2018). PBAT mulch films have been shown to degrade effectively in agricultural soils. Moreover, their degradation may improve soil quality by increasing soil organic carbon and microbial biomass carbon, thereby contributing to soil health (Ma et al., 2023; Wang K. et al., 2025). In freshwater and sediment environments, PBAT undergoes significant changes in both chemical structure and aggregation morphology, indicating that degradation can occur in aquatic systems (Fu et al., 2020). PBAT also exhibits biodegradation in simulated marine environments. The degradation behavior is influenced by several factors, including molecular weight, composition, crystallinity, environmental temperature, humidity, oxygen availability, microbial community composition, and the presence of inorganic fillers (Liu B. et al., 2022; Rüthi et al., 2023). Composting conditions typically promote the most efficient degradation of PBAT because elevated temperature, high humidity, and abundant microbial activity can accelerate enzymatic hydrolysis and microbial mineralization (Santos-Beneit et al., 2023). For example, the degradation behavior of PBAT/reed fiber (RF) films were investigated using Fourier-transform infrared spectroscopy (FTIR) and scanning electron microscopy (SEM). As control, the biodegradation rate of PBAT/RF films reached 90.43% within 91 days (Wang et al., 2025b). Overall, PBAT demonstrates effective biodegradation in soil, aquatic environments, and composting systems. This property helps reduce plastic accumulation and mitigate environmental pollution. However, during degradation, PBAT may generate microplastic particles, and the potential environmental impacts of these particles should be carefully considered in practical applications.

## 5 Blend of PBAT

PBAT integrates the excellent biodegradability of aliphatic polyesters with the relatively high mechanical strength of aromatic polyesters. However, its relatively low stiffness and high production cost remain major limitations for large-scale industrial applications. Compared with rigid biodegradable polyesters such as polylactic acid (PLA) and poly (3-hydroxybutyrate-*co*-3-hydroxyvalerate (PHBV), PBAT exhibits much higher flexibility and elongation at break, but lower tensile strength and Young’s modulus (Patil et al., 2026). In contrast, PCL shows excellent flexibility but relatively low melting temperature and mechanical strength, while polybutylene succinate provides relatively balanced mechanical properties and processability. TPS and starch-based materials are attractive because of their low cost, renewability, and biodegradability (Küster et al., 2025). However, their poor moisture resistance and weak mechanical properties restrict their direct applications. Therefore, blending PBAT with other biodegradable polymers or natural macromolecules is an effective strategy to balance mechanical strength, toughness, biodegradability, processability, and cost. This blending strategy can regulate the microstructure of PBAT-based materials, including chain interactions and interfacial compatibility between different phases. As a result, the mechanical properties, thermal stability, barrier performance, and environmental resistance of PBAT-based materials can be significantly improved (Lai et al., 2020; Pietrosanto et al., 2020; Yoksan et al., 2021). The representative properties of neat PBAT and other biodegradable polymers are summarized in Table 1, while the mechanical properties of PBAT-based composites are listed in Table 2.

**TABLE 1:** Comparison of representative properties of PBAT and other biodegradable polymers.

Polymer	Tensile strength (MPa)	Young’s modulus (MPa)	Elongation at break (%)	T_g_ (°C)	T_m_ (°C)	Main advantages	Main limitations	Ref.
PBAT	18–35	40–100	>500	∼-30	110–130	High flexibility, excellent ductility, good film-forming ability, good processability, biodegradability	Relatively low stiffness and tensile strength; relatively high cost	Garalde et al. (2019), Jian et al. (2020), Küster et al. (2025), Sui et al. (2023), Tang et al. (2024)
PLA	21–70	2000–3,500	<10	50–80	150–180	High stiffness, high tensile strength, good transparency, good processability	Brittle, poor toughness, low elongation at break	Deng et al. (2018), Farah et al. (2016)
PBS	30–40	300–700	100–400	−45 to −10	90–120	Good toughness, good processability, better flexibility than PLA	Lower stiffness than PLA; degradation strongly depends on environmental conditions	de Matos Costa et al. (2020), Muthuraj et al. (2014)
PCL	10–25	200–500	300–1,000	∼-60	55–65	Very flexible, good biodegradability and biocompatibility, low processing temperature	Low melting point, low stiffness, limited thermal resistance	Santos-Beneit et al. (2023), Sousa et al. (2022), Yang B. et al. (2023)
PHBV	25–45	2000–3,500	1–8	−5 to 5	150–176	High stiffness, good biodegradability, bio-based microbial polyester	Brittle, narrow processing window, relatively high cost	Zhao et al. (2023), Zytner et al. (2023)
TPS/starch-based polymers	2–15	50–500	10–150	Variable	No clear T_m_/processing-dependent	Renewable, low cost, rapid biodegradability, high bio-based content	Moisture sensitivity, poor mechanical strength, poor dimensional stability	Yimnak et al. (2020), Lopes et al. (2021), Liu Z. et al. (2025)

**TABLE 2:** Relative changes in the mechanical properties of PBAT blends compared with neat PBAT.

Blend	Tensile			Impact strength	Flexural		Ref.
	Tensile strength	Young’s modulus	Elongation at break		Maximum stress	Flexural modulus	
PBAT30/PLA70	432.3%	3,012.5%	​	​	​	​	Dmitruk et al. (2023)
PBAT19/PLA81	96.6%	2,260.0%	−72.5%	​	​	​	Deng et al. (2018)
PBAT30/PLA70	−84.2%	2,400.0%	−99.0%	​	​	​	Su et al. (2020)
PBAT70/PLA30	−21.1%	200.0%	−75.4%	​	​	​	Su et al. (2020)
PBAT30/PLA70/ESO5	​	1,595.7%	−43.1%	​	​	​	Han et al. (2020)
PBAT25/PLA70/GMA5	176.7%	4,371.1%	−98.8%	41.0%	​	​	Kumar et al. (2010)
PBAT75/PBS25	​	66.5%	−43.0%	​	​	​	de Matos Costa et al. (2020)
PBAT70/PBS30	150%	​	−6.7%	​	​	​	Muthuraj et al. (2014)
PBAT5/PBS95	​	15%	−58.0%	1,200%	​	​	Wu et al. (2019)
PBAT60/PBS40/ADR1	77.5%	196.3%	​	​	​	​	Li et al. (2026)
PBAT5/PBS5/PLA80/nHA5	60.8%	​	−98.7%	​	​	​	Chen et al. (2023)
PBAT75/PCL25	−15.4%	116.7%	−21.8%	​	​	​	Sousa et al. (2022)
PBAT80/PCL20	−27.5%	76.2%	3.9%	​	​	​	Zhang et al. (2025b)
PBAT85/PCL15	61.5%	116.7%	​	​	​	​	Qian et al. (2025)
PBAT70/PHBV30	4%	808.9%	−15.0%	​	300.0%	558.9%	Zytner et al. (2023)
PBAT75/PHBV25	16.9%	573.8%	​	​	​	1,000.0%	Zhao et al. (2023)
PBAT50/PHBV50/V0.1	​	513.3%	−82.8%	​	​	​	Pawar et al. (2015)
PBAT80/PHBV20	−6.25%	400.1%	−12.5%	​	600.0%	​	Wang et al. (2025a)
PBAT90/TPS10	21.1%	​	​	16.3%	14.9%	​	Zhang et al. (2025a)
PBAT60/TPS40/ZA3	−0.5%	20.2%	25.2%	​	​	​	Yimnak et al. (2020)
PBAT80/TPS20/HNT5	−15.0%	66.2%	−53.1%	​	​	​	Dang et al. (2020)
PBAT70/TPS30	−52.9%	​	−41.2%	​	​	​	Cai et al. (2024)
PBAT70/TPS30/TAIC2	−31.3%	​	−53.6%	​	​	​	Cai et al. (2024)
PBAT90/CNC10	−16.7%	47.1%	−35.0%	​	​	​	Morelli et al. (2016)
PBAT/CNC0.02	27.0%	26.0%	37.0%	​	​	​	Lai et al. (2020)
PBAT/CNC0.05	30.8%	10.4%	12.7%	​	​	​	Kim et al. (2023)
PBAT78/CNC-AC12	30.4%	90.1%	−9.1%	​	​	​	Francisco et al. (2022)
PBAT92/CNF8	168.7%	64.6%	−62.2%	​	​	​	Fourati et al. (2021)
PBAT80/MCC20	−55.6%	175.0%	−82.4%	​	​	​	Botta et al. (2021)
PBAT/E-XR1	22.8%	58%	16.0%	​	8.3%	43.0%	Pan et al. (2026)
PBAT/Lingin50	−19.4	767.8%	−44.0%	​	​	​	Xiong et al. (2023)
PBAT/Lignin0.5	32.4%	31.8%	35.1%	​	​	​	Guo et al. (2024)
PBAT/Lingin0.2	−10.5%	10.5%	−9.4%	​	​	​	Kargarzadeh et al. (2024)
PBAT90/Lignin10	6.7%	18.4%	4.4%	​	​	​	Botta et al. (2022)
PBAT90/Lignin10	37.8%	​	−1%	​	​	​	Shorey and Mekonnen (2022)
PBAT99/Lignin0.13/ZnO0.87	−10.7%	−0.4%	37.5%	​	​	​	Wang et al. (2024a)
PBAT90/Lignin10	1%	37.1%	5.1%	​	​	​	Xing et al. (2017)
PBAT/Lingin10	14.7%	​	19.5%	​	​	​	Lee et al. (2026)
PBAT/AL20	−25%	76.5%	−72.4%	​	​	​	Liu Z. et al. (2025)
PBAT90/Lignin10/DCP0.3	​	​	​	​	126.9%	392.1%	Xu H. et al. (2024)
PBAT90/CaCO_3_10	−4.1%	​	−6.2%	​	​	​	Shang et al. (2023)
PBAT70/CaCO_3_30	−42.2%	75.9%	−41.1%	​	​	​	Yu et al. (2025)
PBAT95/Kaolin5	111.1%	−38.2%	​	​	​	​	Venkatesan et al. (2023)
PBAT95/Sumecton5	59.7%	​	−44.2%	​	​	​	Adak and Teramoto (2025)
PBAT30/TPS70/Cu_2_O	−34.8%	−44.4%	37.7%	​	​	​	Bumbudsanpharoke et al. (2023)

### 5.1 PBAT blend with biodegradable plastics

As a biodegradable polymer, PBAT must maintain good biodegradability after blending modification. Therefore, blending PBAT with other biodegradable plastics, such as PLA, PBS, PCL, and PHBV, has become a widely adopted strategy.

#### 5.1.1 PBAT blends with PLA

PBAT and PLA have been extensively explored for biodegradable blends systems because of their complementary mechanical characteristics and favorable degradability. PLA exhibits high stiffness, high modulus, and excellent processability. However, it suffers from inherent brittleness with an elongation at break below 10% (Farah et al., 2016). In contrast, PBAT is a highly ductile polyester, exhibiting an elongation at break exceeding 500% and a relatively low Young’s modulus (Nam et al., 2024). Consequently, blending PLA with PBAT provides an effective approach for balancing stiffness and toughness, enabling the development of biodegradable materials with synergistically enhanced mechanical performance (Zhou et al., 2016). Beyond conventional film applications, recent studies have further expanded the application scenarios of PBAT/PLA blends. For example, Yu et al. investigated PBAT/PLA blends for fused deposition modeling (FDM) 3D printing and reported that all PBAT/PLA samples could be printed smoothly. The incorporation of PBAT slightly improved the ductility of PLA, enhanced thermal stability, and increased crystallinity, indicating the potential of PBAT/PLA blends for additive manufacturing applications (Yu et al., 2024). Dmitruk et al. fabricated PBAT/PLA blends under different temperatures (−20 °C, 0 °C, 20 °C and 40 °C) to tailor their mechanical and processing properties. The results showed that the tensile strength and Young’s modulus of the blends at ambient temperature were improved by 432.3% and 3,012.5%, respectively, compared with neat PBAT. In addition, the prepared composites could absorb more impact energy during accidental collisions, making them promising candidates for thin-walled honeycomb structures in sports helmets (Dmitruk et al., 2023).

**FIGURE 3:**
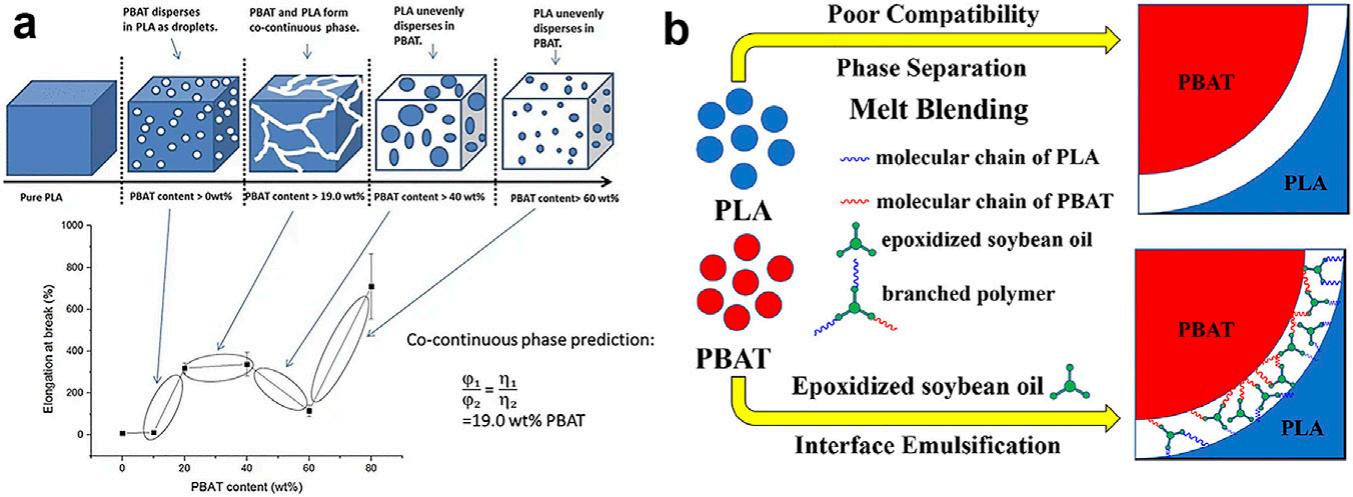
PBAT/PLA blends modified with different compatibilizers. **(a)** Effect of PBAT content on elongation at break (Deng et al., 2018). Copyright 2018, Springer. **(b)** Schematic illustration of the compatibilization mechanism of epoxidized soybean oil (ESO) in PBAT/PLA composites (Han et al., 2020). Copyright 2020, RSC Publications.

A number of studies have demonstrated that the incorporation of PBAT can significantly enhance the toughness of PLA-based systems (Maroufkhani et al., 2021). For instance, Wang et al. prepared PBAT/PLA blend films through solution casting using chloroform as the solvent. The results showed that the toughness of PLA films was greatly improved with increasing PBAT, while acceptable mechanical strength was maintained (Wang et al., 2016). Similarly, Deng et al. prepared PBAT/PLA blends with PBAT ratios from 0/100 to 100/0 and systematically evaluated the influence of PBAT content on mechanical properties (Figure 3a). When the PBAT content reached 19 wt%, the tensile strength and Young’s modulus of the blend were approximately 45 MPa and 1,180 MPa, representing increases of 96.6% and 2,260% compared with neat PBAT (23 MPa and 50 MPa), respectively (Deng et al., 2018). Su et al. further examined PBAT/PLA blends and confirmed that their manufacturability, miscibility, and mechanical properties were strongly dependent on blend composition. Although PBAT can improve the ductility of PLA, poor miscibility and phase separation remain major limitations restricting the overall mechanical performance of uncompatibilized blends (Su et al., 2020).

Despite these advantages, the intrinsic immiscibility between PLA and PBAT remains a major challenge. Thermodynamic incompatibility between the two polymers usually causes phase separation and weak interfacial adhesion, thereby deteriorating the mechanical performance of the blends. To address this issue, multiple compatibilization strategies have been explored, and reactive compatibilization has proven particularly effective. For example, glycidyl methacrylate (GMA) has been widely used as a reactive compatibilizer to enhance interfacial interactions between PLA and PBAT. The tensile modulus increased from 39.06 MPa for neat PBAT to 1746.4 MPa containing 5 wt% GMA, which was attributed to interfacial reactions and the formation of random terpolymers (Kumar et al., 2010). In addition to improving tensile and impact properties, GMA can also contribute to enhanced thermal stability of the blends. In addition to GMA-based compatibilization, *in situ* reactive compatibilization has also been used to regulate PBAT/PLA interfacial structure. Wang et al. reported that this strategy improved the mechanical properties, rheological behavior, and phase morphology of high-toughness PBAT/PLA blends. During reactive blending, the compatibilizer interacts with PLA and PBAT chains, increasing melt elasticity, viscosity, and interfacial compatibility (Wang et al., 2019).

Epoxy-functional chain extenders provide another reliable method for optimizing the compatibility of PBAT/PLA blends (Figure 3b). During melt processing, these additives react with the terminal hydroxyl and carboxyl groups of PLA and PBAT, leading to the formation of branched or slightly cross-linked structures. For example, epoxidized soybean oil (ESO) generates branched molecular architectures and microgels through epoxy–end-group reactions. The incorporation of 5 phr ESO boosts the Young’s modulus by 1,595.7%, greatly improving material toughness. ESO also improves the sustainability of the system because it is derived from renewable resources (Han et al., 2020). Besides ESO, commercial epoxy-functional chain extenders have also been widely investigated for improving the processing stability and long-term performance of PBAT/PLA blends. Joncryl, another widely used epoxy-functional chain extender, can also optimize the rheological and mechanical properties of PBAT/PLA blends. It enhances interfacial compatibilization and promotes phase morphologies, particularly at elevated processing temperatures. Although tensile strength generally decreases with increasing processing temperature, Joncryl-modified blends, especially those prepared with fresh PBAT, exhibit significantly improved elongation at break and impact toughness. Notably, impact strength increases with processing temperature, and the effect is more prominent in Joncryl-containing systems (Altınbay et al., 2025). More recently, Liu et al. demonstrated that enhanced interfacial compatibility can help PBAT/PLA blends maintain higher mechanical properties after physical aging, further confirming the importance of interfacial regulation in improving the long-term performance of PBAT/PLA materials (Liu W. et al., 2024).

#### 5.1.2 PBAT blends with PBS

Blending PBAT with PBS has emerged as an effective strategy for improving the biodegradability of polymer films. Both PBAT and PBS are biodegradable aliphatic polyesters with good processability, but they exhibit different mechanical characteristics. PBS generally possesses higher stiffness and strength, whereas PBAT exhibits superior flexibility and ductility. Therefore, combining these two polymers provides an effective approach for tailoring the mechanical properties of biodegradable materials. Based on this complementary relationship, many studies have focused on the effects of PBS content, phase morphology, and processing conditions on PBAT/PBS blend performance. Fundamental studies have demonstrated that PBS can significantly enhances the stiffness of PBAT-based materials. PBAT/PBS binary blends show pronounced shear-thinning behavior and complex thermos-rheological responses, which are associated with the formation of co-continuous phase morphologies, particularly for blends containing 50 wt% PBAT (de Matos Costa et al., 2020). In addition to mechanical and rheological properties, blend composition and processing conditions also play decisive roles in regulating the microstructure and overall performance of PBAT/PBS blends. In a relevant study, de Beluci et al. prepared PBAT/PBS biodegradable films via extrusion blow molding and found that varying PBS/PBAT ratios significantly affected the water vapor permeability of the films, indicating that composition control is critical for optimizing packaging-related barrier properties (de Beluci et al., 2024).

Apart from conventional melt blending, optimized processing techniques can further improve the interfacial interaction and mechanical performance of PBAT/PBS blends. Muthuraj et al. reported that PBAT/PBS blends prepared by twin-screw extrusion significantly improved tensile strength and elongation at break. Such improvement was attributed to *in situ* ester exchange reactions between the two polyesters during melt processing, leading to the formation of copolyesters and enhanced interfacial compatibility. Nevertheless, conventional twin-screw extrusion only achieves limited modification effects, and the resultant blends still present a distinct two-phase morphology, where PBAT acts as the dispersed phase and restricts the growth of PBS matrix spherulites (Muthuraj et al., 2014). To break through this limitation and further optimize interfacial compatibility and mechanical properties, reactive extrusion has been adopted as an advanced modification method. Wu et al. successfully prepared PBAT/PBS nanoblends using reactive extrusion (Wu et al., 2019). The reactive extrusion process enables PBAT domains to be tightly encapsulated by PBAT/PBS copolymers and uniformly dispersed in the PBS matrix with particle sizes below 100 nm. Remarkably, the addition of only 5 wt% PBAT increased the notched impact strength of PBS by 1,200% and Young’s modulus by 15%. These results demonstrate that reaction-induced nanostructure is an effective approach for optimizing the mechanical performance of PBAT/PBS blends (Wu et al., 2019). Mechanistically, the performance enhancement of PBAT/PBS blends is closely related to interfacial chemical reactions and morphology regulation. During melt blending or reactive extrusion, ester exchange reactions between PBAT and PBS chains can lead to the formation of interfacial copolyester segments. These copolymers effectively improve phase compatibility, reduce interfacial tension, and enhance stress transfer between PBAT and PBS phases, thereby contributing to improved stiffness, toughness, and barrier properties (de Matos Costa et al., 2020; Muthuraj et al., 2014).

Beyond conventional binary systems, the morphology regulation and compatibilization effect of PBS can also be extended to multi-component biodegradable blends. As reported by Chuakhao et al., non-reactive melt-mixed PLA/PBAT/PBS ternary blends exhibit unique interfacial structural characteristics, where PBS microspherulites selectively localize at the PLA/PBAT interface. This interfacial localization allowed PBS to act as a natural compatibilizer, reducing interfacial tension and refining the phase morphology. Moreover, reactive extrusion can further improve the mechanical performance of PLA/PBAT/PBS blends even at relatively high PBS contents, demonstrating the broader potential of PBS for morphology regulation in PBAT-based biodegradable blends (Chuakhao et al., 2024).

In addition to conventional film applications, PBAT/PBS blending also shows considerable potential for functional foaming materials. To optimize the foaming performance and dimensional stability of PBAT-based foams, Li et al. introduced PBS to improve the crystallization behavior and melt strength of PBAT and further incorporated 1 wt% chain extender ADR to enhance interfacial compatibility and inhibit foam shrinkage. The optimized formulation containing 40 wt% PBS and 1 wt% ADR enabled the successful fabrication of uniform PBAT microcellular foams. Compared with pure PBAT foams, the modified foams exhibited a 196.3% increase in Young’s modulus and a 77.5% improvement in tensile strength, providing a feasible strategy for the industrial injection molding of high-performance biodegradable microcellular foams (Li et al., 2026). Furthermore, the combination of ternary polymer blending and inorganic nanofiller modification can further endow PBAT-based materials with specialized functional properties for advanced applications. Chen et al. incorporated nanohydroxyapatite (nHA) with biological activity and osteoconductivity into PLA/PBS/PBAT ternary blends and systematically investigated the synergistic effects of component ratio and nanofiller content on physicomechanical properties. The optimized formulation (80 wt% PLA, 15 wt% PBS, 5 wt% nHA) exhibited a tensile strength of 57.9 MPa, approximately twice that of neat PBAT. Meanwhile, the blend maintains excellent elongation at break and impact strength, making it a promising candidate material for bone graft applications (Chen et al., 2023).

Overall, PBS modification is a versatile and effective strategy to simultaneously improve the stiffness, toughness, and barrier properties of PBAT-based biodegradable polymer systems. Through component complementation, interfacial reaction regulation, and multiscale structure optimization, PBAT/PBS binary blends and related multicomponent systems can achieve comprehensive performance improvement. Nevertheless, precise control of phase morphology and interfacial compatibility remains essential for achieving optimal mechanical performance and functional customization of such biodegradable blends.

#### 5.1.3 PBAT blends with PCL

**FIGURE 4:**
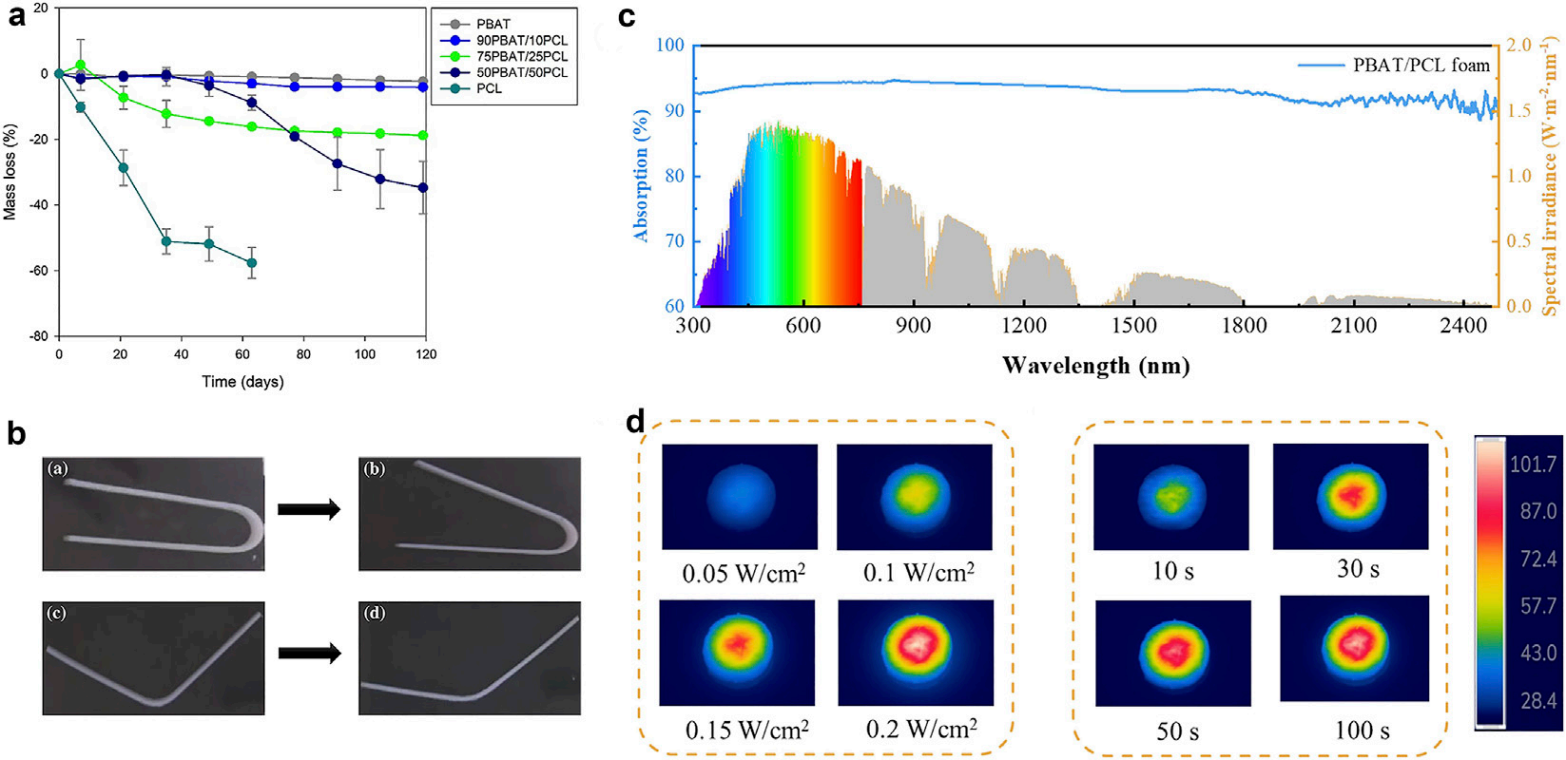
Properties of PBAT/PCL blends. **(a)** Biodegradation of neat PBAT, PCL, and PBAT/PCL blends (Sousa et al., 2022). Copyright 2022, Springer. **(b)** Response process for shape memory recovery (Yang B. et al., 2023). Copyright 2023, Springer. **(c)** Absorption spectra of PBAT/PCL foam ranging from 300 to 2,500 nm (Qian et al., 2025). Copyright 2025, Elsevier. **(d)** Heat transfer images of PBAT/PCL foams (Qian et al., 2025). Copyright 2025, Elsevier.

Compared with PCL, PBAT exhibits a relatively slower degradation rate under environmental conditions, which limits its effectiveness in applications requiring rapid post-use degradation. Therefore, blending PBAT with PCL represents a practical strategy for accelerating biodegradation while largely preserving key mechanical and barrier properties. Previous studies have demonstrated that incorporating PCL can significantly enhance the degradability of PBAT without causing pronounced deterioration in tensile strength, modulus, or permeability (Sousa et al., 2019; Yang B. et al., 2023). As shown in Figure 4a, soil burial tests revealed average mass losses of 2.3% for neat PBAT and 34.7% for a PBAT50/PCL50 blend after 119 days, indicating that the degradation rate of PBAT increased by approximately 14-fold upon blending with PCL. Increasing the PCL content in PBAT/PCL blends markedly improved biodegradability while maintaining tensile strength values comparable to those of neat PBAT. In terms of barrier performance, the incorporation of PCL resulted in decreased oxygen permeability and increased carbon dioxide permeability relative to pure PBAT. This behavior was attributed to the higher solubility of CO_2_ in both PCL and PBAT matrices (Sousa et al., 2022). In 2023, Yang et al. prepared biodegradable PBAT/PCL blends with shape-memory properties (Figure 4b). Bending tests showed that the shape-memory fixation and recovery ratios reached 96% and over 80%, respectively (Yang B. et al., 2023). In addition to conventional films, PBAT/PCL blends have also been explored as functional materials. Qian et al. developed multi-responsive shape-memory PBAT/PCL foams via a one-step foaming and sinter-molding process. When the PCL content was below 15 wt%, the mechanical performance of the foams was significantly improved. Moreover, the resulting PBAT/PCL foams responded to both near-infrared (NIR) light and microwave irradiation. The absorption behavior of carbon nanotube (CNT)-coated PBAT/PCL foams was systematically investigated (Figure 4c), revealing absorbance values exceeding 90% across a broad spectral range of 300–2,500 nm. Upon NIR irradiation at a power of 0.3 W, the foam temperature rapidly increased to 87 °C, which was sufficient to trigger shape recovery, demonstrating the feasibility of using NIR light as an external stimulus for actuating PBAT/PCL-based shape-memory foams (Figure 4d) (Qian et al., 2025).

Additionally, Zhang et al. prepared PBAT/PCL films via twin-screw extrusion and observed a significant enhancement in film stiffness with increasing PCL content. In particular, the Young’s modulus increased by 76.2% compared with that of neat PBAT. In contrast, the oxygen permeability of the blended films was not significantly different from that of pure PBAT. Notably, both CO_2_ and water vapor permeabilities initially decreased and subsequently increased with increasing PCL content, reaching minimum values at a PCL loading of 20 wt% (Zhang et al., 2025a).

#### 5.1.4 PBAT blends with PHBV

**FIGURE 5:**
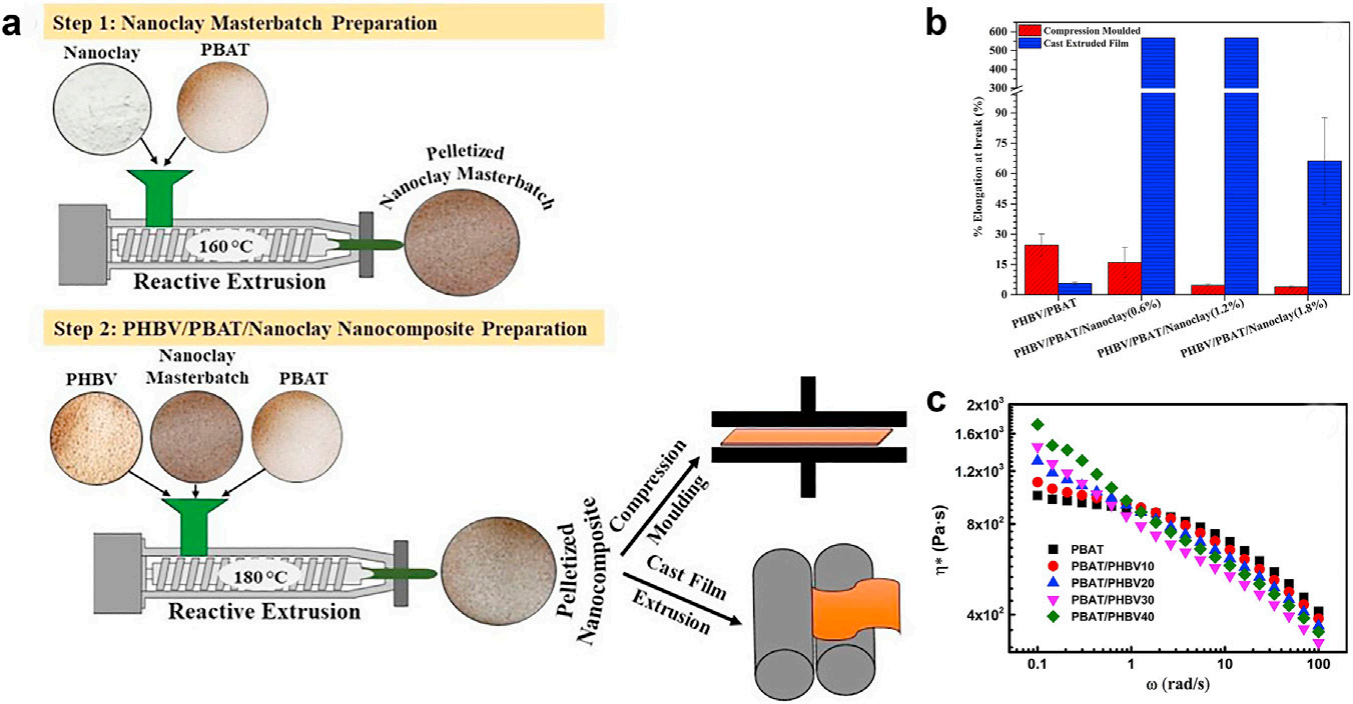
Improvements in the properties by introducing additional functional additives. **(a)** Schematic representation of the experimental scheme for preparing PHBV/PBAT/nanoclay-based strands (Pal et al., 2020). Copyright 2020, Elsevier. **(b)** Elongation at the break of PBAT/PHBV-based compression-molded and cast-extruded films (Pal et al., 2020). Copyright 2020, Elsevier. **(c)** Dynamic rheological properties of PBAT/PHBV blends (Wang et al., 2025a; Wang et al., 2025a) Copyright 2025, MDPI.

PHBV, a biodegradable polyester produced by microorganisms, exhibits excellent biodegradability; however, its inherent brittleness limits its applications. Blending PHBV with PBAT provides an effective approach to overcome this limitation by improving ductility (Zytner et al., 2023). The incorporation of PBAT significantly increased the Young’s modulus of PHBV/PBAT blends by 808.9%. Moreover, as the PBAT content increased, the fracture behavior of the blends transitioned from brittle to ductile, accompanied by a 1,000% increase in the flexural modulus (Zhao et al., 2023). Further improvements in the mechanical performance can be achieved through the introduction of additional functional additives. For example, Pal et al. incorporated Nanomer® I.30T nanoclay (20 wt%) into PBAT using a co-rotating twin-screw extruder. Prior to extrusion, PBAT and nanoclay were manually premixed in a zip-lock bag and subsequently fed into the extruder hopper. The resulting PBAT/nanoclay strands were cooled in a chilled water bath and pelletized (Figure 5a). The incorporation of organically modified nanoclay had a pronounced effect on the properties of PHBV/PBAT blends. The PHBV/PBAT/nanoclay containing 1.2 wt% nanoclay composite film exhibited significantly improved elongation at break compared with the unfilled blend (Figure 5b), while simultaneously showing enhanced barrier properties against oxygen and water vapor (Pal et al., 2020). Similarly, the use of a styrene–methyl methacrylate–glycidyl methacrylate (SMMG) copolymer as a multifunctional reactive compatibilizer promoted interfacial adhesion through the formation of branched or lightly cross-linked PBAT-*b*-PHBV structures. This strategy improved the mechanical properties and also enhanced the storage modulus and melt viscosity (Figure 5c), thereby improving blow-molding processability (Wang et al., 2025a). PHBV/PBAT composites containing 0.1 wt% vermiculite (V) exhibited enhanced tensile modulus, flexibility, and biodegradability (Pawar et al., 2015). Rheological analysis further revealed that the blends showed increased complex viscosity (η*) at low frequencies, while the tan δ values decreased with increasing PHBV content, indicating that PHBV mainly contributed to the elastic rather than the viscous response. These studies show that both reactive compatibilization and nanoparticle reinforcement can improve the properties of PHBV/PBAT biodegradable composites.

### 5.2 PBAT blends with natural macromolecules

Blending PBAT with natural macromolecules provides an effective approach to enhance the sustainability and performance of biodegradable polymer systems. The incorporation of natural macromolecules, such as starch, cellulose, and lignin, increases the bio-based content and degradation rate of the blends, thereby reducing reliance on petrochemical resources. Moreover, these blends may exhibit improved mechanical properties, thermal stability, and gas barrier performance while simultaneously lowering production costs and maintaining good processability.

#### 5.2.1 PBAT blends with TPS

The combination of PBAT and TPS primarily aims to overcome the intrinsic limitations of TPS, including low mechanical strength and high moisture sensitivity, while maintaining the biodegradability and cost advantages of both components (Liu Y. et al., 2025). TPS loading plays a decisive role in tailoring the mechanical performance of PBAT/TPS blends. Zhang et al. found that the tensile strength of TPS/PBAT composites decreased from 13.7 to 3.83 MPa as the TPS content increased from 0% to 50%. However, at a low TPS loading, the flexural and tensile strengths of PBAT/TPS10 increased by 14.9% and 16.3%, respectively, compared with pure PBAT (Zhang C. et al., 2025).

In addition to component proportion, the processing method is another critical factor affecting the dispersion state, interfacial quality, and final performance of PBAT/TPS blends. Zhang et al. explored the influence of different processing routes on modified starch/PBAT films and found that internal mixing for 15 min followed by extrusion and film blowing produced the best overall performance. The optimized films exhibited a tensile strength of 10.5 MPa with good processability and improved thermal stability. The tensile strength remained above 7.09 MPa even when the modified starch content reached 60% (Zhang et al., 2025b). Solid-state shear pulverization (SSSP) also improves the dispersibility, processability, and mechanical strength of the blends. Compared with films produced via conventional melt extrusion, SSSP-processed PBAT/TPS films exhibit improved appearance and mechanical performance, particularly at high starch contents (Lopes et al., 2021). Besides processing routes, blending sequence, plasticizer content, and additive incorporation also influence the morphology and properties of PBAT/TPS blends. Pre-mixing TPS with additives prior to blending with PBAT improves dispersion and compatibility, thereby enhancing the mechanical performance of the blends. Yimnak et al. verified that for PBAT/TPS (60/40) blends modified with 3 wt% zeolite (ZA), the Young’s modulus and elongation at break were increased by 20.2% and 25.2%, respectively, compared with neat PBAT (Yimnak et al., 2020). Further studies have confirmed that processing optimization is also applicable to high-starch-content PBAT systems with challenging modification difficulties (Küster et al., 2025). It indicates that processing optimization enables the fabrication of high-starch, low-cost and high-performance PBAT/starch composite films.

On the basis of processing optimization, nanofillers incorporation represent another effective strategy for improving the compatibility and mechanical properties of PBAT/TPS blends. Various inorganic and organic nanofillers can serve as reinforcing phases to regulate the phase structure of composite systems. Dang et al. adopted halloysite nanotubes (HNTs) to reinforce PBAT/TPS blends, and the results showed that 5 wt% HNT loading increased the Young’s modulus by 66.2%, although the tensile strength and elongation at break decreased to 15.0% and 53.1%, respectively (Dang et al., 2020). Similarly, the addition of cellulose nanofibrils (CNF) promoted a more uniform distribution of TPS within the PBAT matrix, thereby improving the homogeneity of the blends (Fourati et al., 2021).

In addition to physical modification, chemical functionalization can further optimize the interfacial interaction and comprehensive performance of PBAT/TPS blends. Cai et al. prepared crosslinkable TPS by blending corn starch, glycerol, initiator bis(1-(tert-butylperoxy)-1-methylethyl)-benzene and crosslinker 1,3,5-tri-2-propenyl-1,3,5-triazine-2,4,6(1H,3H, 5H)-trione (TAIC). Their results showed that TAIC acted as an effective crosslinker, improving the mechanical properties of the composite films and promoting interfacial compatibility between TPS and PBAT. The enhancement in mechanical performance was mainly attributed to intramolecular crosslinking of TPS induced by TAIC and co-crosslinking at the TPS/PBAT interface, which strengthened interfacial adhesion (Cai et al., 2024). Wongphan et al. introduced reactive additives including nitrite and erythorbate into PBAT/TPS blends for functional modification. These treatments led to smoother microstructures and improved oxygen barrier properties. In addition, they contribute to improved mechanical and barrier performance and may provide additional functionality, such as reducing discoloration in packaged meat products (Wongphan et al., 2023).

#### 5.2.2 PBAT blends with cellulose

Blending PBAT with cellulose is a promising strategy to enhance the mechanical and functional properties of biodegradable polymers, which is applicable to sustainable packaging and agricultural applications. Cellulose fillers can be classified as cellulose nanocrystals (CNC) (Fang et al., 2020), cellulose nanofibrils (CNF) (Li Z. et al., 2021), and cellulose microcrystals (MCC) (Graham et al., 2019). According to their size and morphology, these cellulose fillers can improve PBAT-based composites through different mechanisms, including nanoscale reinforcement, interfacial regulation, and biomass-derived filling.

**FIGURE 6:**
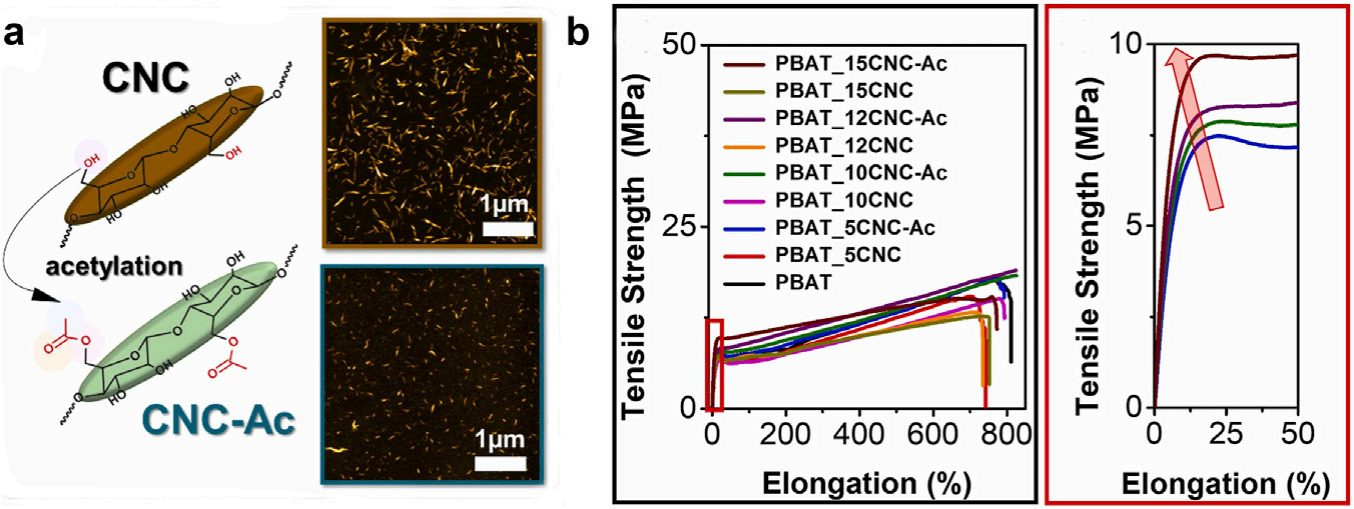
Preparation and properties of PBAT blends with cellulose. **(a)** Chemical scheme of the acetylation reaction and AFM images. **(b)** Mechanical properties of PBAT/CNC-Ac nanocomposite formulations (Francisco et al., 2022). Copyright 2022, Elsevier.

As one of the most widely used nanocellulose reinforcements, CNC have been proven to effectively improve the mechanical performance of PBAT-based composites (Arslan et al., 2023; Dhali et al., 2023; Morelli et al., 2016). For example, Lai et al. reported that the addition of only 0.01 wt% CNC to PBAT increased Young’s modulus by 26%, tensile strength by 27%, elongation at break by 37%, and toughness by 56% compared with neat PBAT (Lai et al., 2020). Kim et al. prepared PBAT/CNC nanocomposites through an *in situ* polymerization strategy and showed that the incorporation of aqueous CNC could substantially enhance the mechanical properties of PBAT. The prepared films, containing only 0.05 wt% CNC relative to the total amount of monomers, exhibited a tensile strength of 71 MPa and an elongation at break of 1,018%, which were reported as among the highest values for PBAT-based composites. Compared with neat PBAT, these values represented increases of 30.8% and 12.7%, respectively, indicating that well-dispersed CNC can act as effective reinforcing nanofillers in PBAT matrices (Kim et al., 2023). Morphological and rheological analyses indicate that the uniform dispersion of nanocellulose in the PBAT matrix effectively inhibits particle aggregation, maintains structural integrity under shear stress, and stabilizes the mechanical properties of compositions during processing (Mohammadi et al., 2021). In addition to dispersion control, surface functionalization provides another route to further improve the reinforcing efficiency of CNC. Further functionalization of CNC can improve its reinforcing efficiency. Francisco et al. modified PBAT with acetylated CNC (CNC-Ac) via solvent casting and hot pressing. Unmodified CNC exhibited an average diameter of 36 ± 6 nm and an aspect ratio (L/d) of 8 ± 2. After acetylation, the resultant CNC-Ac showed a slightly reduced length with a diameter of 38 ± 5 nm and an aspect ratio of 6 ± 1 (Figure 6a). PBAT/CNC-Ac blend with 12 wt% filler exhibited a 30.4% increase in yield strength and a 90.1% increase in Young’s modulus, while maintaining a superior plastic deformation capacity exceeding 650% (Figure 6b) (Francisco et al., 2022). Beyond mechanical reinforcement, CNC can also contribute to the functional performance of PBAT-based films. The incorporation of CNC reduces oxygen permeability in PBAT-based films without compromising moisture or odor barrier properties, which is beneficial for packaging applications (Melendez-Rodriguez et al., 2021).

Apart from CNC, CNF are another important class of cellulose-based reinforcing fillers. In PBAT/TPS blends, CNF can act as both reinforcement agents and interfacial regulators. Fourati et al. incorporated CNF into the TPS phase via twin-screw extrusion. Their results showed that CNF addition improved tensile strength and modulus, with the optimal performance achieved at a loading of 8 wt%. This improvement was associated with a narrower distribution of TPS nodules in the PBAT matrix, indicating that CNF can optimize blend morphology and enhance mechanical properties (Fourati et al., 2021). Esterified CNFs also exhibit excellent interfacial-regulating ability in multicomponent systems. Their amphiphilic characteristics enable a bridge-like effect at the PBAT/TPS interface, thereby improving interfacial compatibility and mechanical properties (Kong et al., 2023).

Besides nanoscale cellulose fillers, microsized MCC derived from natural biomass also show great potential for reinforcing PBAT composites. Both the raw material source and interfacial modification of MCC can influence the comprehensive performance of the final composites. For instance, MCC derived from almond shells increased the elastic modulus of PBAT composites by 175% (Botta et al., 2021). Li et al. further enhanced the interfacial interactions between PBAT and MCC, resulting in improved mechanical performance. Specifically, the puncture load and tearing strength of the PBAT/MCC composite (MCP-1) increased to 25.69 N and 197.61 N/mm, representing increases of approximately 113.7% and 66.4%, respectively, compared with neat PBAT (Li et al., 2023). In addition to almond shells, other cellulose-rich agricultural wastes have also been explored as eco-friendly fillers for PBAT composites. Xylose residue (XR), a low-value agricultural byproduct, contains approximately 61 wt% cellulose. Pan et al. prepared high-performance PBAT composites via multiscale interfacial engineering that combined solvent-free esterification with reactive extrusion. This combined modification effectively overcame the poor compatibility between hydrophilic XR and hydrophobic PBAT. The composite containing only 1 wt% esterified XR showed pronounced performance improvements, with tensile strength, Young’s modulus, flexural strength, and flexural modulus increasing by 22.8%, 58.0%, 8.3%, and 43.0%, respectively. This work provides a green and scalable route for developing biodegradable PBAT composites and highlights the potential of agricultural waste utilization in circular material development (Pan et al., 2026).

#### 5.2.3 PBAT blends with lignin

Lignin, a natural macromolecule derived from plant biomass, possesses unique ultraviolet (UV) absorption, mechanical reinforcement and antibacterial properties (Sadeghifar and Ragauskas, 2020). Lignin-based composite materials can provide multidimensional barrier functions, including oxygen resistance, water vapor barrier properties, UV shielding, flame retardancy, and antibacterial activity. Owing to these advantages, they have been widely explored for food and pharmaceutical packaging, agricultural protection, construction materials, and other fields requiring high barrier performance, serving as sustainable alternatives to conventional petroleum-based materials (Han et al., 2025). One of the primary advantages of incorporating lignin into PBAT is the enhancement of mechanical performance. The addition of lignin can increase the tensile strength and elongation at break while improving the toughness and flexibility of PBAT composites. These improvements are attributed to the uniform dispersion of lignin within the PBAT matrix, which facilitates stress transfer and polymer chain interactions (Lin et al., 2021; Xiong et al., 2022). For example, a PBAT film containing 1 wt% lignin exhibited a tensile strength of 15.87 MPa and an elongation at break of 602.21% (Lin et al., 2021).

However, pristine lignin usually shows poor compatibility with PBAT because of its strong polarity, rigid aromatic structure, and tendency to aggregate. Therefore, chemical modification and nanoscale regulation have been developed to improve lignin dispersion, interfacial adhesion, and mechanical reinforcement in PBAT/lignin composites. Esterified lignin has been used to improve the compatibility between lignin and PBAT. Xiong et al. developed strong, tough, and cost-effective PBAT/esterified lignin films, showing that chemical modification of lignin can promote better dispersion and interfacial interaction in the PBAT matrix while reducing material cost (Xiong et al., 2023). The PBAT/lignin composite film containing 50 wt% esterified lignin (P/EL50) exhibited a high tensile strength of 24.7 MPa and an elongation at break of 494.7%, outperforming many previously reported PBAT composite films with high lignin loading. Moreover, its Young’s modulus reached 557.37 MPa, representing an increase of 767.8% compared with neat PBAT. Guo et al. demonstrated that incorporating 0.5 wt% sodium alginate-stabilized lignin nanoparticles (SLNP) into PBAT increased the yield strength, tensile strength, and elongation at break by 32.4%, 31.8%, and 35.1%, respectively (Guo et al., 2024). Similarly, lignin nanoparticles have been reported as promising bio-based additives for PBAT films because their nanoscale size and improved dispersibility can enhance mechanical and packaging-related properties (Kargarzadeh et al., 2024).

**FIGURE 7:**
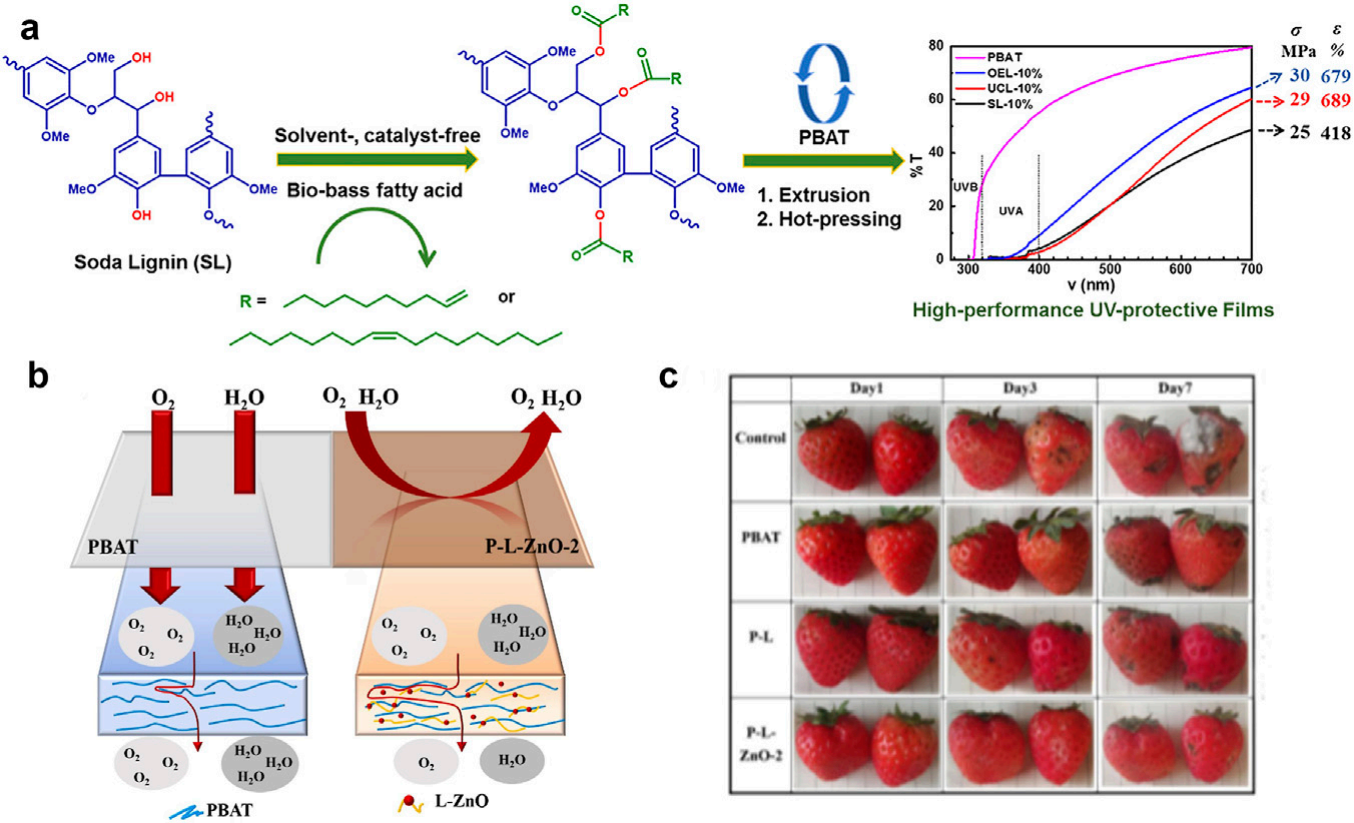
UV protection, barrier properties, and antibacterial properties of PBAT/lignin composites. **(a)** Schematic of the preparation and UV-vis characterization of PBAT/lignin composites (Xing et al., 2017). Copyright 2017, ACS Publications. **(b)** Schematic illustration of the tortuous pathway of O_2_ and H_2_O (Wang S. et al., 2024). Copyright 2024, Elsevier. **(c)** Pictures of the storage process of strawberries (Wang S. et al., 2024). Copyright 2024, Elsevier.

Apart from mechanical enhancement, lignin can also improve the UV resistance and light stability of PBAT composites. The inherent UV absorption ability of lignin protects PBAT-based materials from ultraviolet-induced degradation, which is particularly beneficial for agricultural films and outdoor packaging applications (Botta et al., 2022). Xing et al. observed that films containing 10 wt% lignin provided nearly complete UV protection across the entire UV spectrum. This protection persisted even after 50 h of UV exposure while transparency was maintained or slightly enhanced (Figure 7a) (Xing et al., 2017). Such UV resistance is beneficial for applications exposed to long-term sunlight, including agricultural films and outdoor packaging materials. To further enhance compatibility and durability, lignin nanoparticles can also be combined with bioinspired surface modification. Xing et al. further developed bioinspired melanin-like polydopamine-coated lignin nanoparticles to form UV-blocking core–shell lignin–melanin nanoparticles (LMNPs) with improved compatibility and durability. Films containing 0.5-5 wt% LMNPs blocked nearly all UV-A and more than 80% of UV-B radiation while retaining appreciable optical transparency (Xing et al., 2019).

Furthermore, lignin modification can endow PBAT films with improved barrier and antibacterial properties for food packaging applications. In addition to mechanical reinforcement and UV protection, lignin incorporation can improve the water, oil, and oxygen barrier properties as well as the antibacterial properties of PBAT films, enhancing their potential use in food packaging applications (Shorey and Mekonnen, 2022). Wang et al. prepared PBAT-lignin-ZnO (P-L-ZnO) composite films and demonstrated that the lignin-ZnO (L-ZnO) component maintained the tensile strength and thermal stability of the films while enhancing their hydrophobicity, barrier properties, and antibacterial performance (Wang S. et al., 2024). Compared with neat PBAT, the oxygen transmission rate (OTR) and water vapor transmission rate (WVTR) of P-L-ZnO-2 decreased by 26.7% and 15.0%, respectively. The enhanced hydrophobicity and tortuous diffusion pathways created by L-ZnO hindered the diffusion of O_2_ and H_2_O, thereby improving barrier performance (Figure 7b). These composite films were also used to evaluate strawberry preservation during storage. After 1 week at room temperature, strawberries packaged with the P-L-ZnO-2 composite film showed no mildew and maintained better appearance compared with other samples (Figure 7c).

Chemical modification can also effectively address the inherent drawbacks of biomass-derived kraft lignin, including high molecular weight, poor processability, and inferior compatibility with polymer matrices. Lee et al. verified that phenolated lignin (PL) can substantially improve the poor interfacial compatibility between pristine lignin and biodegradable polymers. At 10 wt% PL, the composite exhibited a tensile strength of 28.12 MPa, which was comparable to that of neat PBAT, while elongation at break and toughness increased by 14.7% and 19.5%, respectively, reaching 1,040% and 208 MJ/m^3^. At a filler loading of 20 wt%, PL achieved homogeneous dispersion and favorable interfacial adhesion within the PBAT matrix, resulting in a 4.6% improvement in toughness compared with neat PBAT. Life cycle assessment (LCA) and techno-economic analysis further demonstrated that PBAT-PL composites showed lower global warming potential and product cost than 17 conventional PBAT-based composites. These results suggest that PL is a promising bio-based filler with high loading capacity and excellent UV-shielding performance for biodegradable polymer blends (Lee et al., 2026). Similarly, Liu fabricated mulch films by hot pressing using PBAT and alkali lignin (AL) as biodegradable raw materials. The optimal overall properties of PBAT/AL mulch films were obtained at an AL loading of 20 wt%. After prestretching treatment, the composite film exhibited a tensile stress of 22.41 MPa and an elongation at break of 252.35%. The outstanding mechanical performance was attributed to hydrogen bonding interactions between AL and PBAT molecular chains. Meanwhile, the PBAT/AL20 composite film showed favorable oxygen permeability, antibacterial activity, and low light transmittance. Outdoor field degradation tests further revealed that PBAT/AL20 exhibited excellent biodegradability, with a mass loss rate of 84.27% within 180 days. This study provides a sustainable strategy for designing high-performance agricultural mulch films and promotes the development of eco-friendly alternatives to conventional polyethylene (PE) mulch films (Liu Z. et al., 2025). In addition to films, lignin has also been incorporated into PBAT foam materials. Beyond conventional films, lignin has also been incorporated into PBAT foam materials. Xu et al. fabricated PBAT/lignin composite foams and reported that lignin incorporation provided a low-cost biomass-based strategy for preparing biodegradable foams with favorable foaming behavior and mechanical properties (Xu H. et al., 2024). Compared with neat PBAT, the flexural strength and flexural modulus of PBAT/lignin composite foams increased by 126.9% and 392.1%, respectively.

Overall, lignin-based modification of PBAT can improve material sustainability and functional performance through multiple mechanisms, including interfacial reinforcement, UV absorption, antioxidant activity, barrier enhancement, antibacterial functionality, and cost reduction.

### 5.3 PBAT blends with inorganic materials

The incorporation of inorganic materials into PBAT-based composites provides an effective strategy for improving mechanical performance, reducing material costs, and expanding their potential applications.

#### 5.3.1 PBAT blends with calcium carbonate

Among the various inorganic fillers, calcium carbonate (CaCO_3_) has been widely investigated because of its low cost and reinforcing capability. Shang et al. demonstrated that CaCO_3_ modified via gas–solid fluidization enabled PBAT/CaCO_3_ composites to maintain good overall performance even at filler loadings as high as 30 wt% (Shang et al., 2023). Surface modification can further improve filler-matrix interactions. Liu et al. reported that titanate-coupling-agent-modified CaCO_3_ significantly enhanced the mechanical and thermal properties of PBAT/CaCO_3_ films. In addition to increased tensile strength and thermal stability, the modified composites exhibited improved water resistance and reduced water vapor permeability. These improvements were attributed to the increased crystallization temperature and crystallinity induced by the modified CaCO_3_, which reinforced the PBAT matrix (Liu et al., 2023). Moreover, solid-state shear grinding (S^3^M) technology has been shown to promote the uniform dispersion of nano-CaCO_3_ in PBAT/PLA matrices, thereby improving compatibility and mechanical performance. The resulting films exhibited high tensile strength and elongation at break, indicating their suitability for environmentally friendly packaging applications (Jia et al., 2024). Beyond conventional reinforcement, CaCO_3_ has also been used to develop PBAT-based films with shape-memory behavior. Yu et al. further fabricated CaCO_3_/PBAT composite films with shape-memory performance via melt blending followed by hot pressing. The incorporation of CaCO_3_ significantly improved the tensile modulus, shape-recovery stress, and thermal stability of PBAT. For the composite film containing 30 wt% CaCO_3_, the tensile modulus and shape-recovery stress reached 100.6 ± 3.1 MPa and 3.26 MPa, respectively, representing increases of 75.9% and 37.0% compared with neat PBAT (Yu et al., 2025).

#### 5.3.2 PBAT blends with clay-based fillers

**FIGURE 8:**
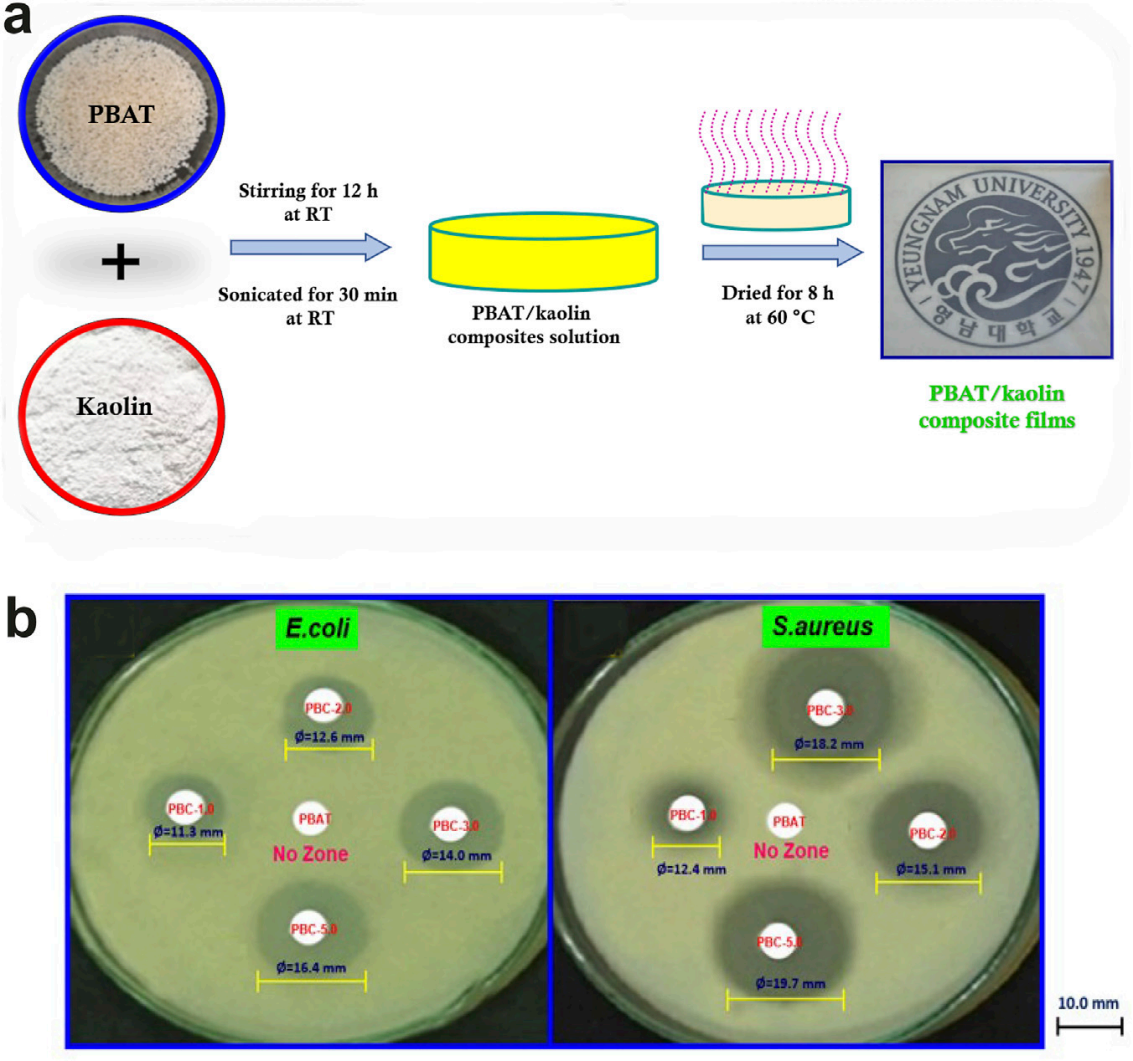
The preparation and properties of PBAT blend with clay-based fillers. **(a)** Fabrication diagram for PBAT/kaolin clay biocomposites. **(b)** Antimicrobial properties of PBAT/kaolin clay biocomposites against *Escherichia coli* and *Staphylococcus aureus* (Venkatesan et al., 2023). Copyright 2023, MDPI.

Kaolin clay is another inorganic filler that can enhance both the structural and functional properties of PBAT composites. Venkatesan et al. incorporated kaolin clay into PBAT matrices via solution mixing and drop casting (Figure 8a), which significantly enhanced mechanical strength and thermal stability. PBAT/kaolin biocomposites prepared via solution casting exhibited a tensile strength of 37.6 MPa, representing an increase of 111.1% compared with virgin PBAT (18.3 MPa) (Venkatesan et al., 2023). Concurrently, the oxygen and water vapor transmission rates were markedly reduced. In addition, PBAT/kaolin composites demonstrated antibacterial activity against *Staphylococcus aureus* and *E. coli*. As the kaolin loading increased from 0 to 5.0 wt%, inhibition zone diameters against *E. coli* increased from 10.0 to 16.4 mm, while those against *Staphylococcus aureus* increased from 10.0 to 19.7 mm (Figure 8b) (Venkatesan et al., 2023).

**FIGURE 9:**
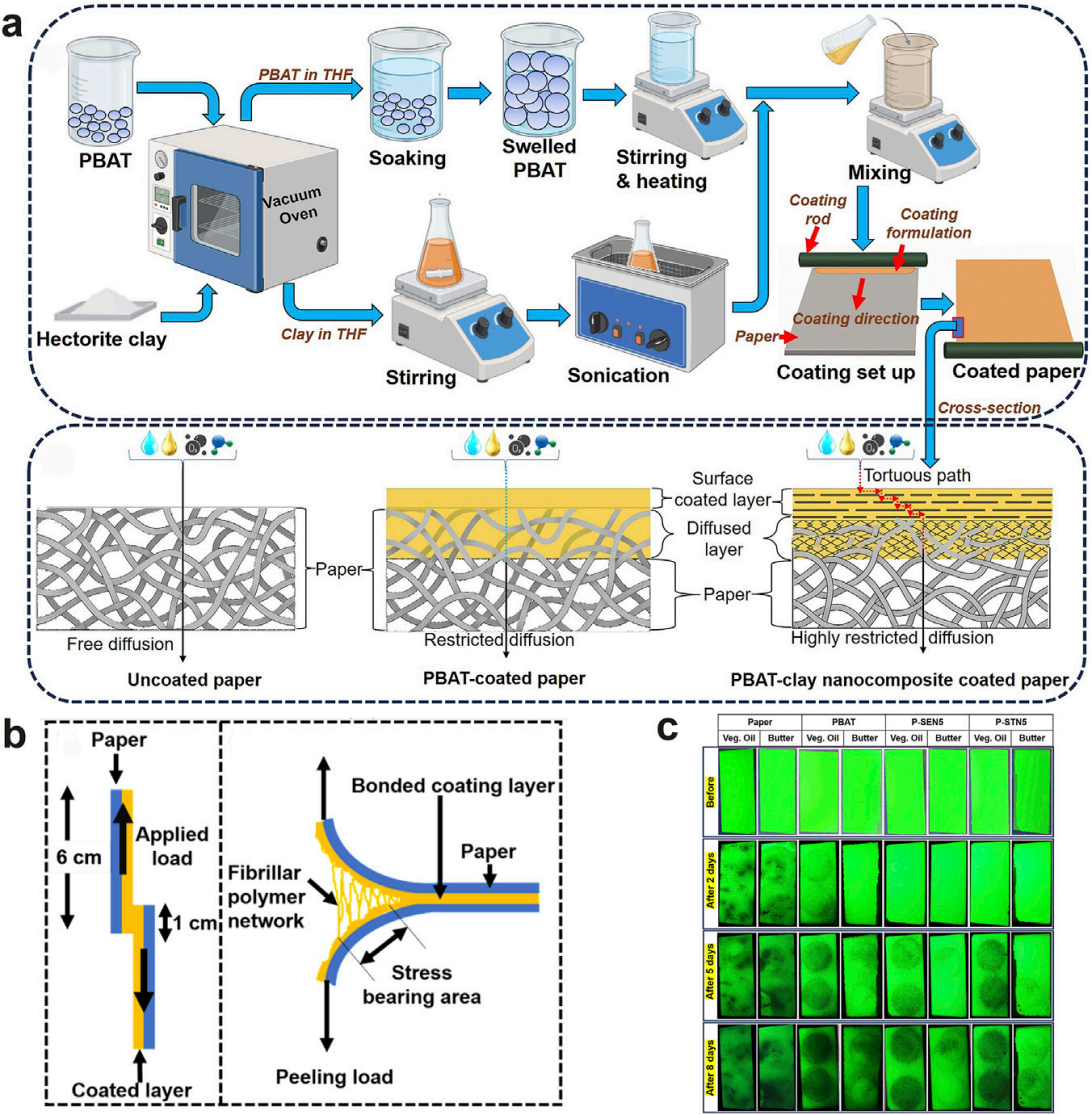
**(a)** Schematic of the preparation and diffusion of water, oil, oxygen, and water vapor in the PBAT–clay nanocomposite. **(b)** Schematic of lap shear strength and the testing of the peeling action of heat-sealed coated paper. **(c)** Photograph of grease permeability testing at 60 °C for uncoated and coated papers (Adak and Teramoto, 2025). Copyright 2025, Elsevier.

In addition to kaolin-based film reinforcement, synthetic hectorite clay has been explored in PBAT-based coating systems for sustainable paper-based packaging. Adak and Teramoto introduced an eco-friendly strategy to improve paper-based packaging by applying a PBAT/synthetic hectorite clay nanocomposite coating (Figure 9a). PBAT-coated paperboard composites exhibited strong self-adhesion without the addition of external adhesives. Adhesion performance was evaluated through shear testing (Figure 9b). The oil repellency of the coated papers was further examined using vegetable oil and butter as model substances. Butter penetrated only the uncoated paper within 2 days, while all coated papers remained impermeable (Figure 9c). These results indicate that PBAT/clay nanocomposite-coated paper can improve oil resistance in paper-based packaging (Adak and Teramoto, 2025). Compared with conventional polymer-coated paper, PBAT/hectorite clay coatings provide a more sustainable strategy for improving adhesion and grease resistance while maintaining the biodegradability of the packaging system.

#### 5.3.3 PBAT blends with copper oxide nanoparticles

While blending PBAT with TPS improves biodegradability, it may reduce mechanical and barrier properties. The incorporation of copper oxide nanoparticles (CuONPs) provides an effective strategy to compensate for these drawbacks while introducing antibacterial functionality. Bumbudsanpharoke et al. reported that the addition of 2 wt% CuONPs increased the elongation at break of PBAT films by 37.7% (Table 1). The PBAT/TPS-CuO bionanocomposites also exhibited antibacterial activity, indicating their potential application in food packaging (Bumbudsanpharoke et al., 2023).

## 6 Application of PBAT

**FIGURE 10:**
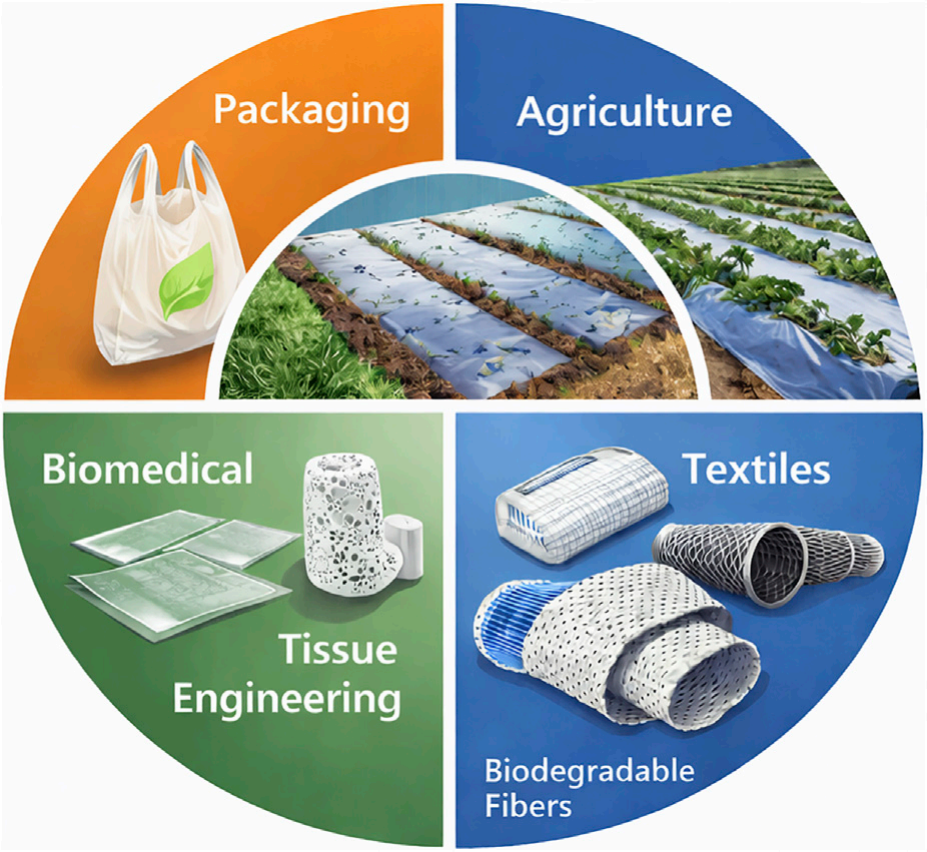
Application overview of PBAT.

The mechanical properties, biodegradability, and compatibility of PBAT with other materials make it a promising material for promoting sustainable development. Consequently, PBAT has been applied in packaging, agriculture, and biomedical engineering. The overall performance of PBAT can be further improved through modification and blending, which not only broadens its application scope but also enhances its functionality in specific applications (Figure 10).

### 6.1 Mulching film

The large-scale and long-term use of PE mulching films in agriculture has caused substantial environmental problems, mainly due to the accumulation of film residues in soil. These residues, often referred to as microplastics (MPs), represent a persistent source of pollution that negatively affects soil health and agricultural productivity (Hou et al., 2019). The presence of MPs can alter soil microbial community structures, interfere with metabolic functions, and influence the biosynthesis of essential cellular processes and secondary metabolites (Wu et al., 2022). The impact of PE mulching film residues is not limited to microbial communities; it can also significantly affect soil bulk density, porosity, and hydrophobicity (Qi et al., 2020). These changes in soil properties may further influence crop yield and soil quality. In addition, the presence of MPs in agricultural soils has been associated with reduced crop yields and potential risks to food security (Khalid et al., 2023).

PBAT-based mulch films are being widely explored as sustainable alternatives to conventional non-biodegradable films. These films can degrade in soil after the growing season, eliminating the need for labor-intensive film removal and reducing soil pollution (Liu B. et al., 2022; Wang K. et al., 2025). PBAT mulching films can degrade effectively under field conditions, thereby reducing the accumulation of MPs in soil (Convertino et al., 2024). At the same time, the use of PBAT mulching films can increase soil organic carbon and microbial biomass carbon, which contributes to improved soil health and crop productivity (Wang K. et al., 2025). In addition, compared with conventional PE mulching films, PBAT mulching films have a marked effect on the compositional structure and functional roles of soil microbial consortia, elevating microbial functional activity and community-level diversity (Liu L. et al., 2022; Liu X. et al., 2025). However, despite their environmental advantages, the long-term effects of PBAT mulching films require further investigation. Some studies have indicated that PBAT films may generate MPs during degradation, and the long-term effects of these particles on soil and plant health remain unclear (Wang K. et al., 2024; Zhou et al., 2023). Therefore, comprehensive environmental risk assessments are necessary when promoting the large-scale use of PBAT mulching films (Xu Z. et al., 2024; Zhang et al., 2022).

### 6.2 Packaging

Traditional petroleum-based plastics have been extensively used in packaging industry owing to their low cost and excellent performance in terms of durability, mechanical strength, lightweight characteristics, and electrical and thermal insulation properties (Filho et al., 2021). These plastics provide effective protection and preservation in food packaging. However, the large-scale use of plastic materials has led to severe environmental challenges, with packaging waste accounting for a substantial proportion of global plastic waste (Ncube et al., 2020). In response to these concerns, PBAT has attracted increasing attention in the packaging industry for the production of compostable materials, including food containers and shopping bags (Ghasemlou et al., 2024). Owing to its biodegradability, PBAT can decompose under industrial composting conditions, thereby reducing the environmental burden associated with persistent plastic waste (Itabana et al., 2024).

The degradation behavior of PBAT/PLA blends varies significantly under different environmental conditions and is closely related to factors such as material thickness, the presence of additives, and the degradation environment (Musioł et al., 2018). In addition, the degradation efficiency of PBAT can be further enhanced through enzyme optimization, thereby facilitating the breakdown under natural environmental conditions (Li et al., 2025). These characteristics provide PBAT with clear advantages in reducing packaging-related plastic pollution. Furthermore, the application of PBAT blended with esterified lignin in paper-based coatings significantly improves the mechanical and barrier properties of the material, highlighting their potential as high-performance materials for food-contact applications (Shorey and Mekonnen, 2022).

Despite these advantages, the widespread application of PBAT in packaging still faces several challenges. The production cost of biodegradable plastics remains relatively high, the required degradation conditions are often strict, and industrial infrastructure for large-scale processing is still limited. Nevertheless, with continued technological innovation and improvements in industrial supply chains, biodegradable plastics are expected to evolve from environmentally friendly alternatives into mainstream packaging materials, contributing to global carbon neutrality goals (Shi et al., 2025).

In addition to environmental benefits, PBAT-based composite films can provide functional advantages for food preservation. Olonisakin et al. reported that the incorporation of lignin into PBAT films effectively reduced color changes and delayed spoilage in packaged food. After 24 days of storage, food wrapped in conventional films exhibited pronounced discoloration and extensive bacterial growth, whereas food packaged with PBAT/lignin composite films showed only minor color changes and no visible bacterial proliferation (Olonisakin et al., 2023). These results highlight the potential of PBAT-based composite films to preserve food quality and prolong shelf life by limiting moisture and oxygen ingress.

### 6.3 Tissue engineering

The application of PBAT in tissue engineering has received growing attention. Neat PBAT scaffolds prepared using various fabrication methods, such as solvent evaporation and electrospinning, have shown favorable characteristics for bone tissue engineering (Arslan et al., 2016). PBAT possesses good mechanical stability and a fibrous morphology; however, its biological activity is relatively low, which limits its direct application in tissue engineering. The biological activity of PBAT can be significantly improved by combining it with extracellular matrix (ECM) materials. For example, electrospun PBAT microfiber membranes coated with decellularized bone ECM can support osteogenesis and show potential for applications in tissue engineering and regenerative medicine (Karakaya et al., 2022). The combination of PBAT with the conductive polymer polypyrrole (PPy) has also been reported to be effective in promoting nerve cell differentiation. PBAT/PPy composites can support the adhesion and differentiation of neuroblastoma cells, suggesting potential applications in neural tissue engineering (Granato et al., 2018). In addition, hollow porous microfiber (HPMF) scaffolds fabricated from PLA/PBAT have been investigated for wound healing applications. The porous architecture of PLA/PBAT HPMFs was constructed via the dissolution of polyvinyl alcohol in an aqueous medium. *In vivo* studies on wound healing showed that the loaded composite scaffolds significantly promoted wound closure by the 13th day (Liu Y. et al., 2024).

### 6.4 Textiles

In the textile industry, increasing attention to sustainability has driven the development and utilization of biodegradable polymers as alternatives to conventional synthetic fibers (Naeimirad et al., 2023). Incorporating PBAT into fibers can reduce environmental pollution and align with the global trend toward green and circular textiles. PBAT can be processed through melt spinning and electrospinning to produce fibers with tunable diameters and morphologies suitable for various textile products (Li X. et al., 2021).

PBAT-based electrospun nanofibrous membranes have been designed to combine super hydrophobicity, breathability, and mechanical robustness. The incorporation of silica nanoparticles enhances infrared reflectivity and thermal regulation, providing comfort and protection in specialized wearable applications (Aijaz et al., 2024). Similarly, PBAT/PHBV/TiO_2_ nanofiber membranes exhibit both biodegradability and antibacterial functionality, achieving inhibition rates above 98% against *E*. *coli* and *S*. *aureus* while maintaining excellent mechanical performance (Xu et al., 2022).

PBAT has also been used to develop waterproof and breathable composite membranes through structural and surface modifications. By combining PBAT with hemp-based nonwoven substrates and hydrophobic silica coatings, composite textiles with high tensile strength, moisture permeability, and water resistance have been obtained, indicating potential applications in degradable medical and protective fabrics (Gao et al., 2023). PBAT has also been utilized to produce biodegradable antimicrobial gauzes via dry-spinning technology, which enables large-scale fiber production with high tensile strength and elongation. After loading with tannic acid, these PBAT fiber tows demonstrated significant antibacterial activity against common pathogens, expanding the potential of PBAT in medical and hygienic textiles (Wang Q. et al., 2025). In addition to fiber production, PBAT-based hot-melt adhesives modified through side-chain engineering have demonstrated improved adhesion strength, flexibility, and hydrolysis resistance, facilitating their use in fabric lamination and textile finishing (Mao et al., 2025).

Overall, PBAT exhibits considerable versatility in textile applications, ranging from functional and protective garments to medical and technical fabrics. Its degradability, adaptability, and compatibility with various modification strategies make it an attractive material for advancing sustainable textile manufacturing and reducing polymer waste generation.

## 7 Conclusion

As a typical biodegradable polyester, PBAT combines flexibility, mechanical strength, and eco-friendly degradability, positioning it as a viable substitute for conventional petroleum-derived plastics. Its well-balanced molecular architecture, consisting of flexible aliphatic segments and rigid aromatic units, provides both ductility and structural stability, enabling its widespread use in sectors such as packaging, agriculture, biomedical engineering, and textiles.

Blending PBAT with other biodegradable polymers (e.g., PLA, PBS, and PHBV) or natural macromolecules such as starch, cellulose, and lignin has been demonstrated to enhance its processability, mechanical properties, and barrier performance, while preserving its inherent biodegradability. In addition, the incorporation of inorganic fillers and nanomaterials can further improve thermal stability and functional properties of PBAT, thereby expanding its potential in high-value sustainable materials.

PBAT exhibits favorable biodegradability under soil, composting, and aquatic conditions, which helps reduce persistent plastic residues in the environment. However, several challenges remain, including incomplete degradation under certain conditions, the potential generation of microplastics during degradation, and the economic feasibility of large-scale production.

Future research should explore green and efficient catalytic systems for PBAT synthesis, optimizing copolymer compositions to balance mechanical strength and degradation rates, and advancing recycling strategies such as enzyme-assisted or chemical recycling strategies to achieve closed-loop material utilization. Furthermore, the design of PBAT-based multifunctional composites integrating properties such as antibacterial activity, UV resistance, and controlled degradation may further expand their practical applications. With continued advances in material chemistry and industrial processing technologies, PBAT is expected to play an important role in the transition toward a sustainable, circular polymer-based economy.
